# Design, synthesis and biological evaluation of 2-((4-sulfamoylphenyl)amino)-pyrrolo[2,3-d]pyrimidine derivatives as CDK inhibitors

**DOI:** 10.1080/14756366.2023.2169282

**Published:** 2023-01-19

**Authors:** Bo Yang, Yanni Quan, Wuli Zhao, Yingjie Ji, Xiaotang Yang, Jianrui Li, Yi Li, Xiujun Liu, Ying Wang, Yanping Li

**Affiliations:** Institute of Medicinal Biotechnology, Chinese Academy of Medical Sciences, Peking Union Medical College, Beijing, China

**Keywords:** Cell cycle-dependent kinase inhibitor, 2-((4-sulfamoylphenyl)amino)-pyrrolo[23-d]pyrimidine derivatives, pancreatic ductal adenocarcinoma, anti-proliferative activity, oral pharmacokinetics

## Abstract

To explore the potential use of CDK inhibitors in pancreatic ductal adenocarcinoma (PDAC) therapy, a series of novel 2-((4-sulfamoylphenyl)amino)-pyrrolo[2,3-d]pyrimidine derivatives was designed, synthesised, and investigated for inhibition on both CDK kinase activity and cellular proliferation of pancreatic cancer. Most of new sulphonamide-containing derivatives demonstrated strong inhibitory activity on CDK9 and obvious anti-proliferative activity in cell culture. Moreover, two new compounds suppressed cell proliferation of multiple human pancreatic cancer cell lines. The most potent compound **2g** inhibited cancer cell proliferation by blocking Rb phosphorylation and induced apoptosis via downregulation of CDK9 downstream proteins Mcl-1 and c-Myc in MIA PaCa-2 cells. CDK9 knockdown experiment suggests its anti-proliferative activity is mainly mediated by CDK9. Additionally, **2g** displayed moderate tumour inhibition effect in AsPC-1 derived xenograft mice model. Altogether, this study provided a new start for further optimisation to develop potential CDK inhibitor candidates for PDAC treatment by alone or combination use.

## Introduction

Cancer is a major public health problem worldwide. Pancreatic ductal adenocarcinoma (PDAC) is one of the most lethal common malignances and ranks fourth in the United States and seventh worldwide among leading causes of cancer-related deaths^1^. Gemcitabine with fluorouracil-based chemotherapies is the main treatment option for advanced PDAC^2^. New small molecule targeted therapy including of EGFR, tyrosine kinase, and PARP inhibitors, and immunotherapy such as PD-1 inhibitor might also be used for patients with PDAC^3^^,^^4^. However, delayed diagnosis, deficiency of effective treatment, and chemoresistance led to a low survival rate of less than 10% for PDAC^5^^,^^6^. Therefore, innovative approaches are urgently needed for PDAC patients.

**Figure 1.**
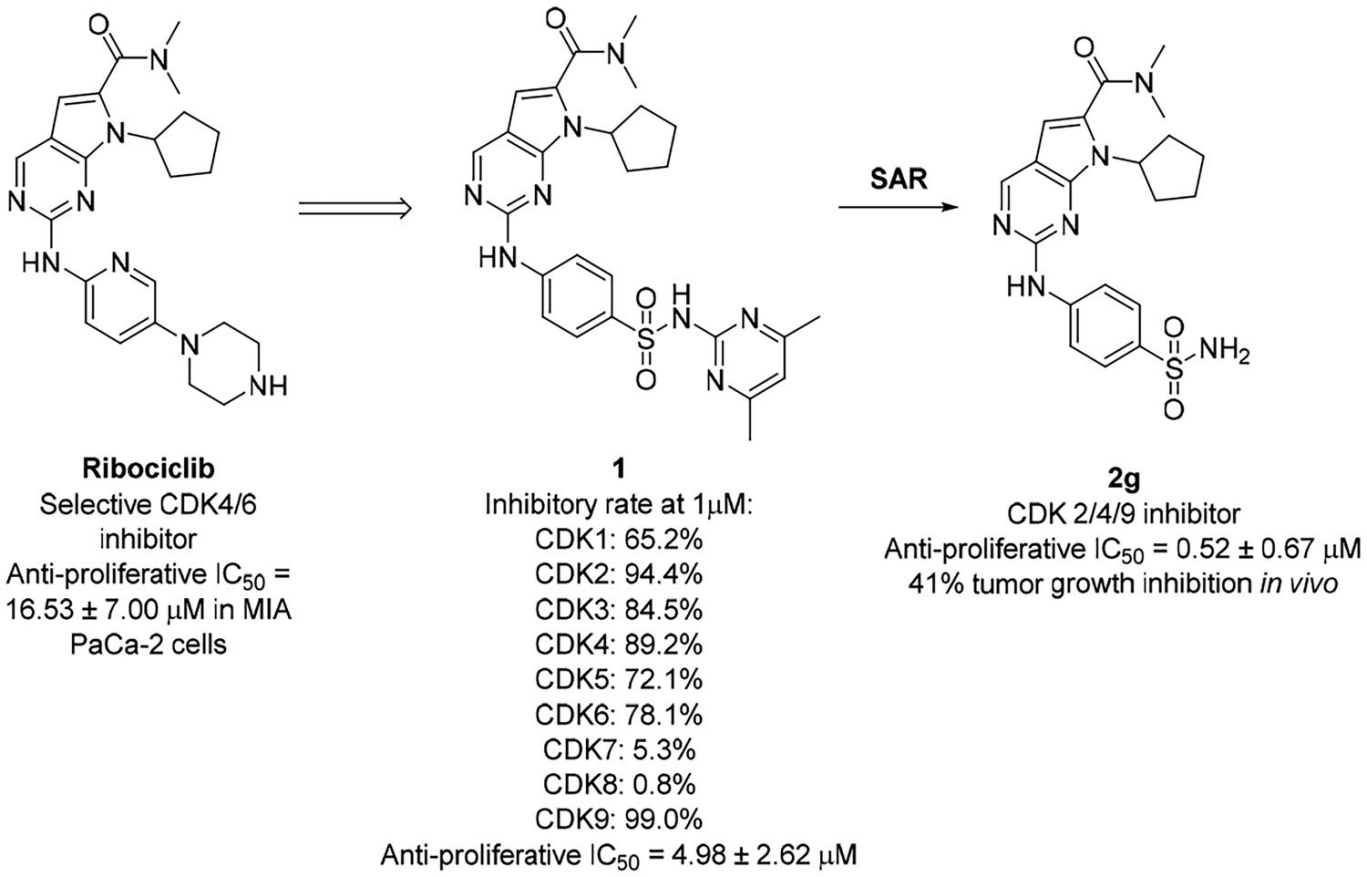
Discovery of 2-((4-sulfamoylphenyl)amino)-pyrrolo[2,3-d]pyrimidine derivatives as CDK inhibitors.

Cyclin-dependent kinase (CDK) is a family of serine-threonine kinases, and consists of about 20 kinases^7^. Each CDK interacts with the specific cyclin to form a CDK-cyclin complex to produce enzyme activity. In addition to the cell-cycle CDKs (CDK1, 2, 4, 6, 7) that directly promote cell cycle progression, some transcriptional CDKs (CDK7, 8, 9) play an important role as regulator of transcription. Even though CDKs have been extensively studied in the past decades, their roles in the cell cycle and other related processes are not yet fully understood. Dysregulated CDKs have been linked to cancer initiation and progression^8^. Thus, it is not surprising that CDKs and their regulators are identified as drug targets in various human cancers^9^. For instance, three CDK4/6 inhibitors (palbociclib, ribociclib, and abemaciclib) have been approved by the FDA for the treatment of advanced oestrogen receptor (ER)-positive breast cancer^10^. Although few clinical evidence of CDK inhibitors was shown in PDAC, the emerging role of CDKs in the pathobiology of this dreadful cancer indicated the therapeutic potential to target CDKs as a novel treatment strategy for PDAC^11–15^. Recently, Sebti’s group also revealed a link between CDK hyperactivation and mutant *Kras* dependency through comparative phosphoproteomics and CRISPR/Cas9 gene editing, and consequently found that knocking out the CDK1, 2, 7, and 9 was as effective as knocking out *Kras* in mutant *Kras*-driven pancreatic cancer^11^. *Kras* mutation is known a major driver and a desirable drug target in pancreatic cancers. Moreover, tumours that harbour *Kras* mutations are highly aggressive, invasive, metastatic, and associated with poor prognosis^16–18^. A very high rate of activating mutations in *Kras* (>90%) exists in most human cancers of pancreas including the earliest stage of pancreatic intraepithelial neoplasia, common *Kras* mutations in pancreatic ductal adenocarcinoma are G12D, G12C, and G12V^19^^,^^20^. Besides of direct inhibit on KRAS protein, targeting RAS effectors or synthetic lethal partners appeared to be an alternative opportunity to treat *Kras*-driven cancers^21^^,^^22^. Therefore, CDK-targeting therapy could be exploited to efficaciously abrogate pancreatic cancer. There are a lot of studies necessary to further evaluate the promise of CDK-inhibitors in highly malignant PDAC. Here, we reported the design, synthesis, and structure-activity relationship (SAR) of a series of new 2-((4-sulfamoylphenyl)amino)-pyrrolo[2,3-d]pyrimidine derivatives as CDK inhibitors against PDAC (Figure 1). Initial mechanism of action and *in vivo* efficacy study confirmed the potential of **2g** as a lead compound to develop new approaches for treatment of PDAC in future study.

## Results and discussion

### Chemistry

**Scheme 1.**
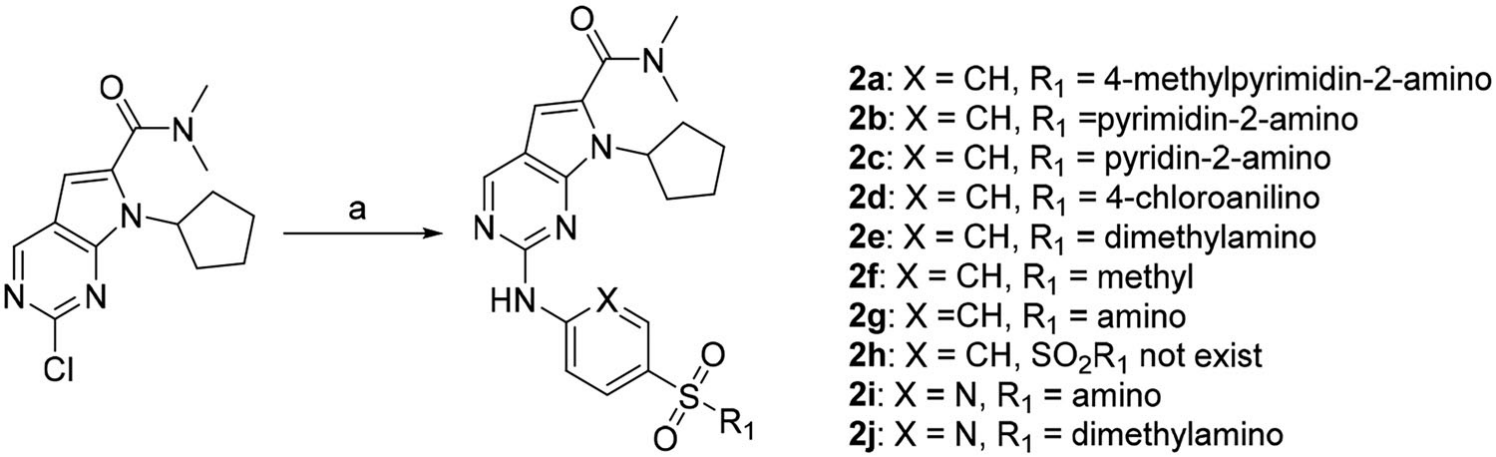
Synthesis of 2-((4-sulfamoylphenyl)amino) substituted derivatives. Reagents and conditions: (a) arylamine, Xantphos, Pd_2_ (db)_3_, Cs_2_CO_3_, DMF, 110 °C, microwave irradiation.

**Scheme 2.**
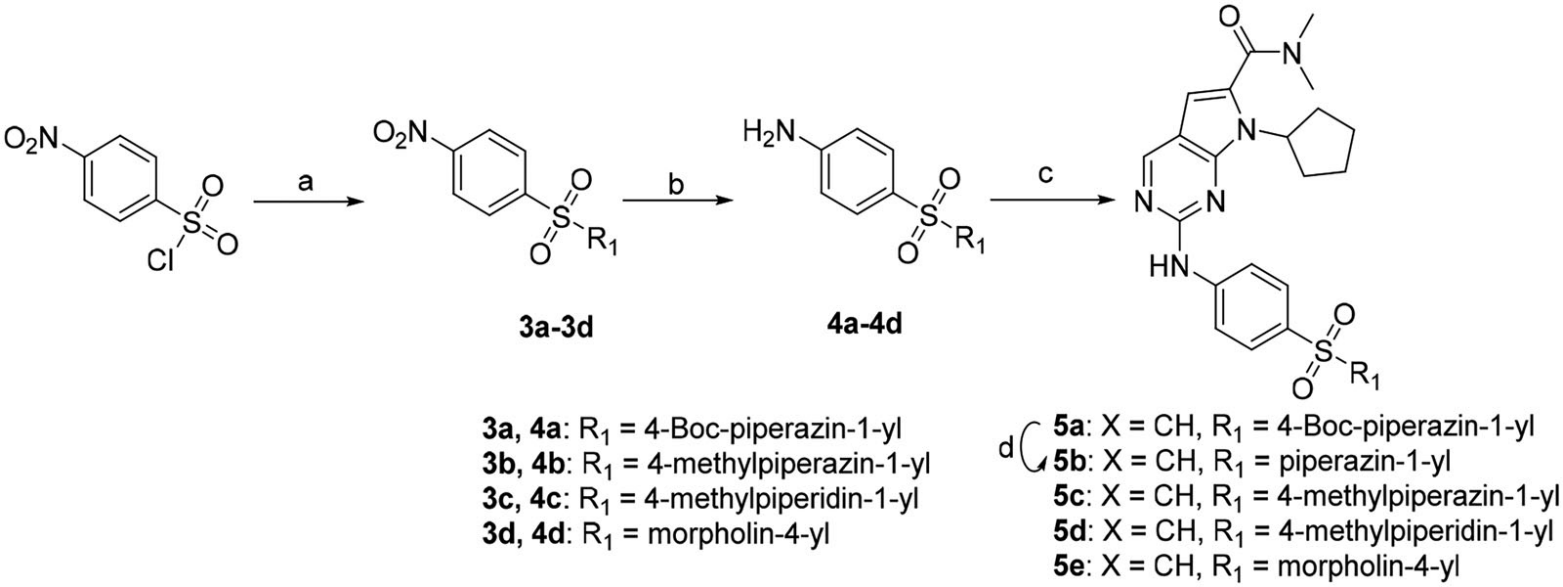
Synthesis of 2-((4-sulfamoylphenyl)amino) substituted derivatives. Reagents and conditions: (a) K_2_CO_3_, DCM,RT; (b) Pd/C, Methanol; (c) 2-chloro-7-cyclopentyl-N,N-dimethyl-7H-pyrrolo[2,3-d]pyrimidine-6-carboxamide, Xantphos, Pd_2_ (db)_3_, Cs_2_CO_3_, DMF, 110 °C, microwave irradiation; (d) 2 M hydrochloride in Methanol.

We previously reported a series of 6-anilinocarbonyl-substituted ribociclib derivatives with stronger anti-proliferative activity than ribociclib in pancreatic cancer cell cultures^23^. These more potent derivatives contain the anilino group instead of dimethylamino group at C6-position of pyrrolo[2,3-d]pyrimidine in the chemical structure of ribociclib. Meanwhile, we were attracted by compound **1** (Figure 1), which contains a distinct moiety of N-pyrimidine benzenesulfonamide in C2-substituent of pyrrolo[2,3-d]pyrimidine, in view of its higher selectivity to CDK9 than CDK4/6. **1** also displayed higher *in vitro* anti-proliferative activity than ribociclib in pancreatic cancer MIA PaCa-2 cell culture. To our knowledge, Wang et al reported anticancer activity of N-alkyl-benzenesulfonamide ribociclib derivatives as CDK9 inhibitor in non-small-cell lung cancer^24^. This appeared to be a new chemical starting point to identify lead compound with novel mechanism of action and strong potency against PDAC cell proliferation. To understand the role of 4,6-dimethyl-pyrimidine in the benzenesulfonamide moiety of compound **1**, we firstly designed and synthesised 2-((4-sulfamoylphenyl)amino)-pyrrolo[2,3-d]pyrimidine derivatives **2a**–**2j** by bioisosteres replacement and fragment cleavage strategy. The synthetic route of **2a**–**2j** was shown in Scheme 1. The classical Pd-catalyzed coupling was performed using commercial available 2-chloro-7-cyclopentyl-N,N-dimethyl-7H-pyrrolo[2,3-d]pyrimidine-6-carboxamide and aromatic amine as start materials at 110 °C under microwave irradiation to afford title compounds in the yield of 16%∼49%. In addition, several N-heterocycle benzenesulfonamide derivatives **5b**–**5e** were designed and synthesised by the method shown in Scheme 2. Starting from 4-nitrobenzenesulfonyl chloride, traditional acylation and subsequent hydrogenation reduction of nitro group to amine were performed to give key 4-sulfonylaniline intermediates **4a**–**4d** through intermediates **3a**–**3d**. **4a**–**4d** were used lately for coupling with 2-chloro-7-cyclopentyl-N,N-dimethyl-7H-pyrrolo[2,3-d]pyrimidine-6-carboxamide by same method as Scheme 1 to afford the corresponding intermediate **5a** and final products **5c–5e**. Removal of Boc protection from **5a** in the presence of hydrochloride or trifluoroacetic acid gave title compound **5b**.

**Scheme 3.**
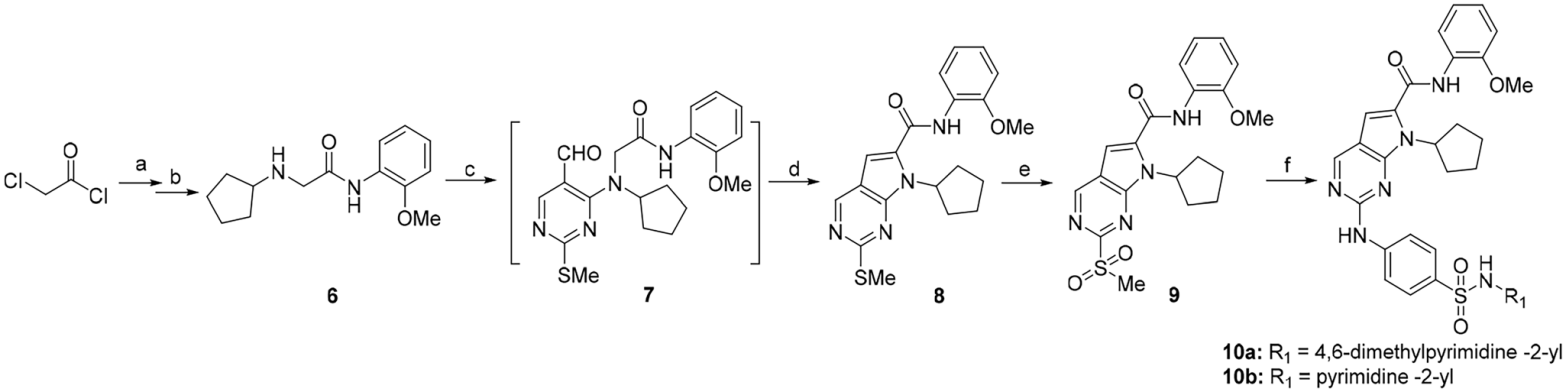
Preparation of 2-((4-(*N*-(pyrimidin-2-yl)sulfamoyl)phenyl)amino)-7*H*-pyrrolo[2,3-*d*]pyrimidine derivatives. Reagents and conditions: (a) 2-methoxyphenylamine, DIPEA, acetonitrile, 0 °C; (b) cyclopentylamine, acetonitrile;(c) 4-chloro-2-(methylthio)pyrimidine-5-carbaldehyde, DIPEA, acetonitrile, MW, 80 °C; (d) Cs_2_CO_3_, MW, 120 °C, 30 min, acetonitrile; (e) m-CPBA, DCM; (f) N-(4-(N-(4,6-dimethylpyrimidin-2-yl)sulfamoyl)phenyl)formamide or N-(4-(N-(pyrimidin-2-yl)sulfamoyl)phenyl)formamide, Cs_2_CO_3_, DMSO, 85 °C

Alternatively, considering that replacing dimethylamino group of ribociclib with aniline increased anti-proliferative activity in previous study, we therefore designed and synthesised new derivatives **10a** and **10b** by introducing the aniline group into C6-substituent in 2-((4-sulfamoylphenyl)amino)-pyrrolo[2,3-d]pyrimidine. The synthetic route shown in Scheme 3 is similar to the previously reported method^23^. Briefly, the secondary amine intermediate **6** synthesised from 2-chloroacetyl chloride and specific amines by a one-pot two-step reaction was reacted with commercial available 4-chloro-2-thiomethylpyrimidine carboxaldehyde in the presence of N,N-diisopropyl ethylamine (DIPEA) to give intermediate **7**, which further underwent the cyclisation reaction to provide pyrrolo[2,3-d]pyrimidine **8** in the presence of caesium carbonate by heating at 120 °C under microwave irradiation. The oxidation of **8** in the presence of m-CPBA in dichloromethane (DCM) afforded intermediate **9** in the yield of 57%. Finally, the aromatic nucleophilic substitution reaction of key intermediate **9** with N-(4-(N-(4,6-dimethyl-pyrimidin-2-yl)sulfamoyl)phenyl)formamide or N-(4-(N-(pyrimidin-2-yl)sulfamoyl)phenyl)formamide in the presence of Cs_2_CO_3_ gave title compounds **10a** and **10b**, respectively.

### Biological evaluation

#### In vitro antiproliferative activity screen in MIA PaCa-2 cells

We screened these new derivatives with GCTB as positive drug in *Kras* mutant human pancreatic cancer MIA PaCa-2 cells. As shown in Table 1, *in vitro* anticancer activity of corresponding derivatives (**2a** and **2b**) became weaker (IC_50_ > 10 µM) than compound **1** (IC_50_ = 4.98 µM) when the number of methyl substituent on the pyrimidine ring. The biological activity was further decreased after the pyrimidine was substituted with pyridine (**2c**, IC_50_ = 18.80 µM). It should be mentioned that poor solubility of compound **2b** and **2c** might exacerbate decreasing of *in vitro* efficacy. Replacement of dimethylpyrimidine with para-chlorophenyl group (**2d**) maintained similar activity (IC_50_ = 6.23 µM) to compound **1**. This indicated that lipophilic terminal of C2-substituent is beneficial to anticancer activity when sulphonyl group was linked with aromatic ring. Interestingly, when we made a stepwise break down the sulphonamide moiety until sulphonamide was removed from compound **1**, corresponding compounds **2e**, **2f**, and **2h** displayed gradually decreased activity comparing to **1**. Surprisingly, nearly ten-fold enhanced potency than **1** was observed for compound **2g** (IC_50_ = 0.52 µM) which has a simple unsubstituted amino group of R_2_. However, further replacing the C2-phenylamino group of compounds **2e** and **2g** with pyridine-2-amino led to almost loss of activity of compounds **2i** and **2j**, respectively. In addition, comparable and even stronger anti-proliferative activity (IC_50_ = 7.8 0 ∼ 0.94 µM) than compound **1** was observed when R_2_ group is saturated heterocycle (**5b**–**5e**). Meanwhile, higher potency of compound **5b** than **5c**–**5e** suggests the role of free NH in the piperazine as hydrogen-bond donor to the biological activity. Alternatively, ten-fold decreased anticancer activity was observed for 6-anilinocarbonyl-substituted derivatives **10a** and **10b** comparing to **1** in this work.

**Table 1.** Anti-proliferative activity of 2-((4-sulfamoylphenyl)amino)-pyrrolo[2,3-d]pyrimidine derivatives in pancreatic cancer MIA PaCa-2 cell culture. 
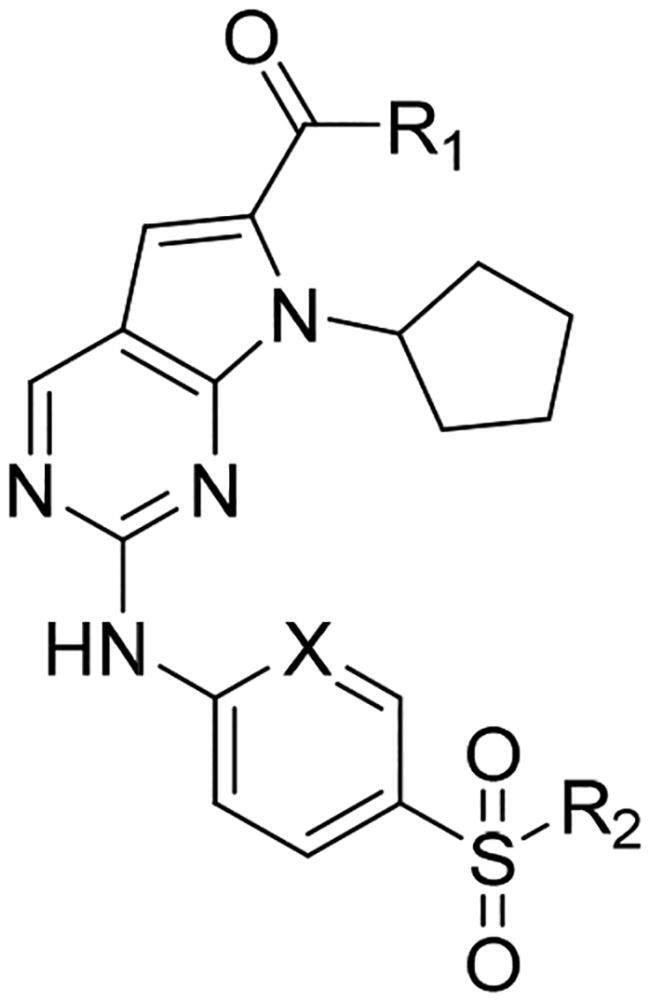

Cpd.	R_1_	R_2_	X	IC_50_ (µM) in MIA PaCa-2^a^	CDK9 inhibitory rate (%) @ 50nM^b^
**1**	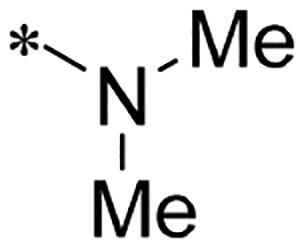	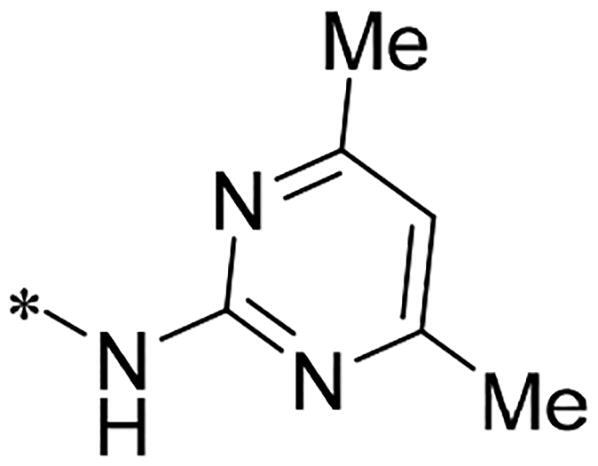	CH	4.98 ± 2.68	98.6
**2a**	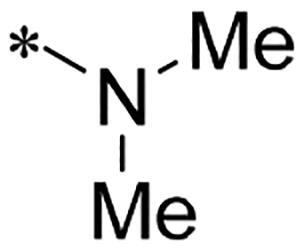	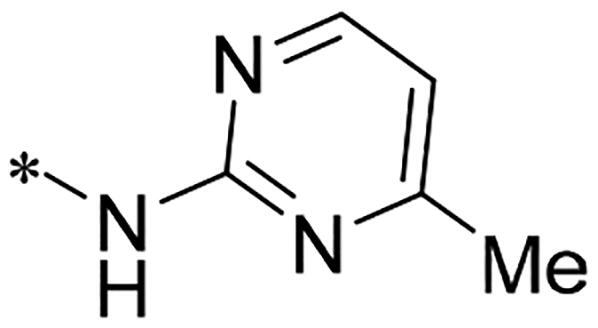	CH	13.00 ± 0.37	100.3
**2b**	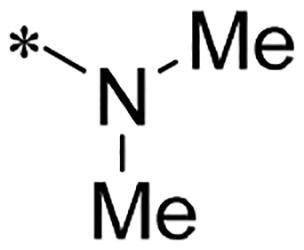	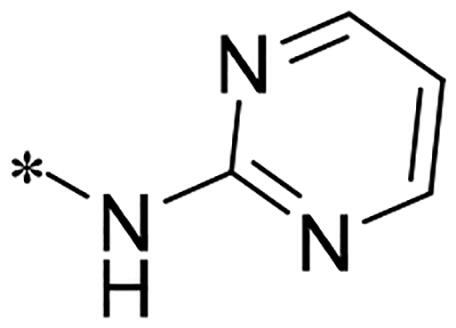	CH	14.35 ± 0.21	98.7
**2c**	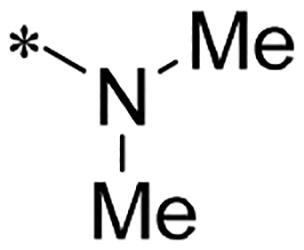	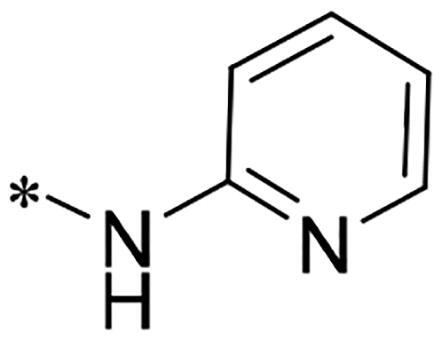	CH	18.80 ± 5.54	99.1
**2d**	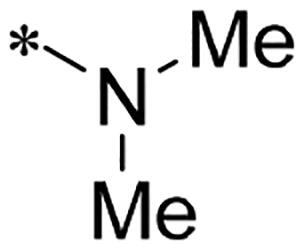	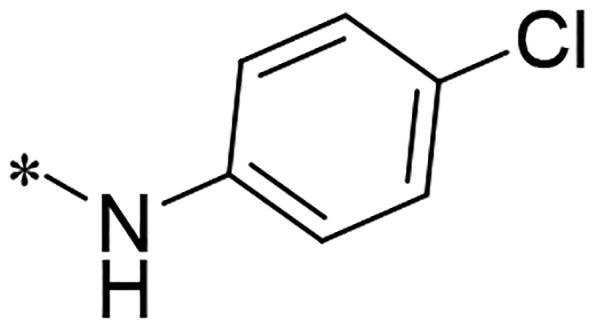	CH	6.23 ± 1.25	91.8
**2e**	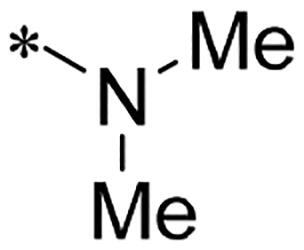	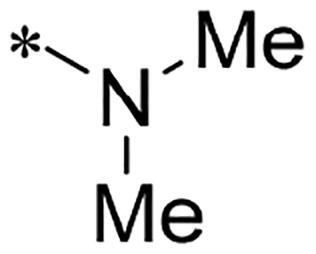	CH	10.50 ± 0.74	89.3
**2f**	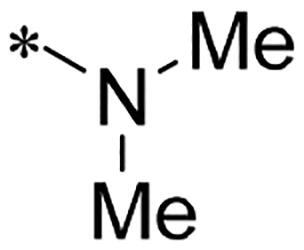	*−CH_3_	CH	18.90 ± 2.121	96.5
**2g**	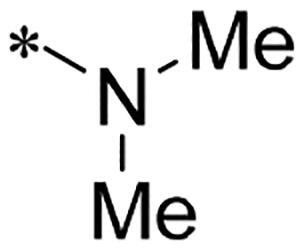	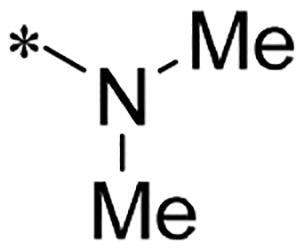	CH	0.52 ± 0.67	97.8
**2h** ^c^	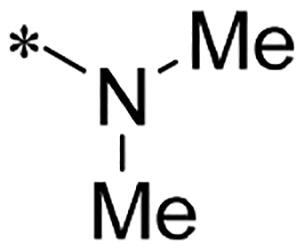	-SO_2_-R_2_ not exist	CH	45.05 ± 16.64	93.2
**2i**	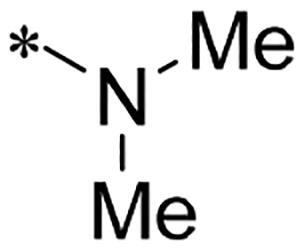	*−NH_2_	N	>100	27.4
**2j**	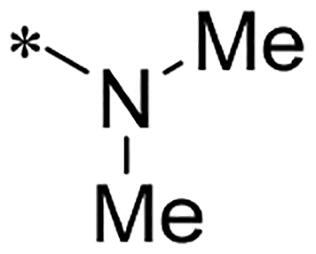	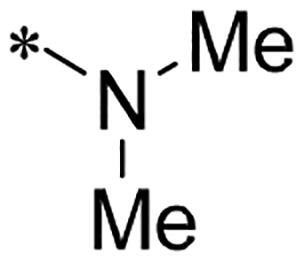	N	69.13 ± 15.51	25.8
**5b**	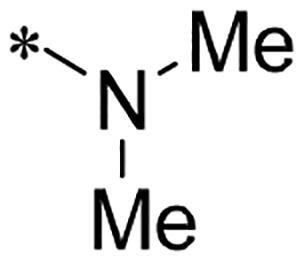	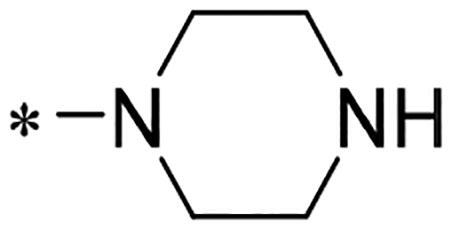	CH	0.94 ± 0.25	98.2
**5c**	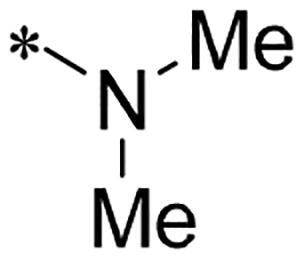	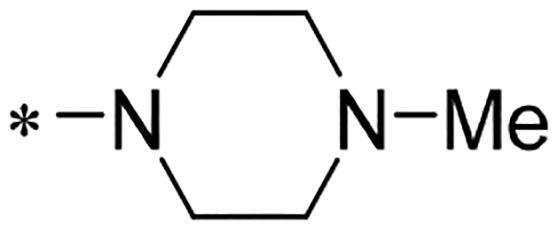	CH	7.80 ± 1.04	99.1
**5d**	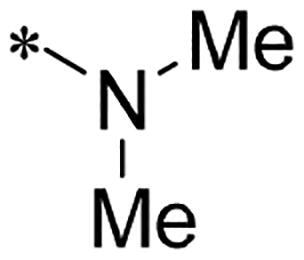	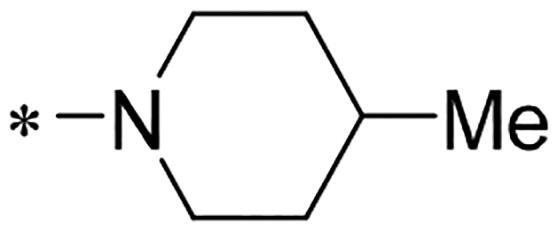	CH	2.85 ± 0.52	80.6
**5e**	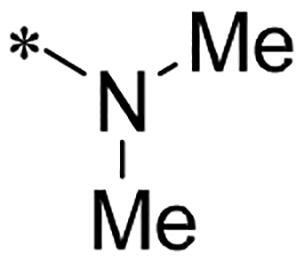	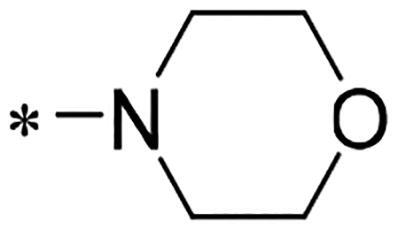	CH	4.35 ± 1.61	81.8
**10a**	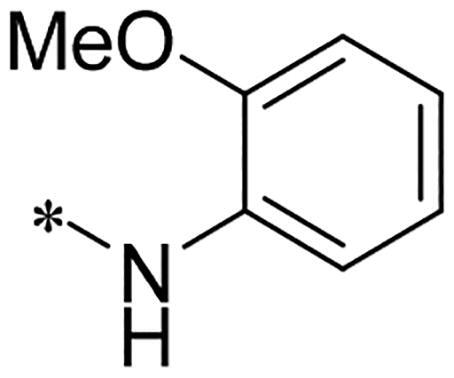	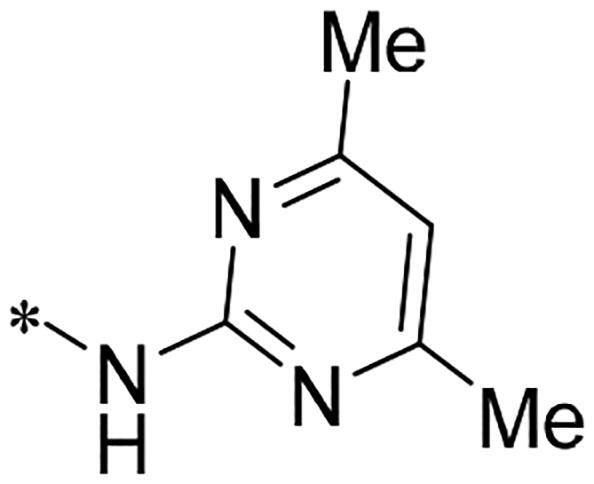	CH	40.76 ± 3.26	−3.1
**10b**	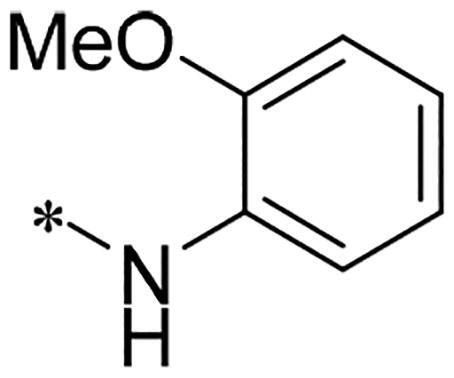	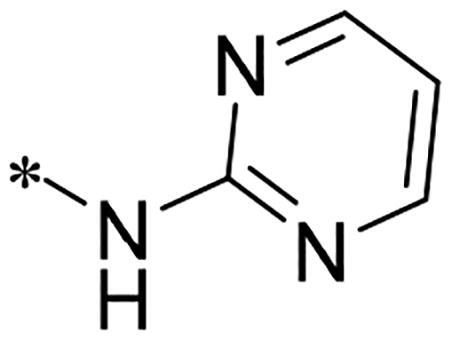	CH	80.76 ± 15.99	54.4
**Ribociclib**				16.53 ± 7.00	16.9
**GCTB**				1.04 ± 0.27	

^a^Cell viability was determined after 72 h drug exposure to cells. Compounds **2b**, **2c**, and **5c** precipitated in cell culture medium during incubation.

^b^Test compounds were screened against on CDK9/cyclinT1 by Lance Ultra Assay with ATP concentration at Km.

^c^compound **2h** was previously reported^23^.

#### CDK protein kinase assay

In previous study, we found that CDK4 inhibitory activity of compound **1** is obviously weaker than highly selectively CDK4 inhibitor ribociclib, but compound **1** exhibited higher *in vitro* anti-proliferative activity compared to ribociclib. CDK kinase screening indicated that compound **1** possessed of the most potent inhibition on CDK9 than other CDK kinase. It led us to speculate that inhibition of CDK9 contributes much to the efficacy of compound **1** against PDAC cell growth. Thus, we firstly screened the CDK9 enzymatic activity inhibitory rate of new derivatives at concentration of 50 nM in a biochemical assay. As shown in Table 1, most of new sulphonamide derivatives possessed of significantly inhibitory activity (>80%) on CDK9. Moreover, these CDK9 inhibitors exhibited higher anti-proliferative activity in MIA PaCa-2 cells than non-CDK9 inhibitors, such as pyrrolo[2,3-d]pyrimidine derivatives with 2-aminopyridine side chain (**2i** and **2j**) or 6-anilinocarbonyl side chain (**10a** and **10b**). Low CDK9 inhibitory potency of compound **2i** (27%) and **2j** (25%) is consistent with the report that pyridin-2-amino moiety is key determinant of high selectivity to CDK4/6^25^. In previous study CDK4/6 inhibition effect was less impaired when dimethylamino group of ribociclib was replaced with *o*-anisidine. However, such a change from **1** to **10a** caused the loss of CDK9 inhibition activity. In general, terminal of C2-substituent of pyrrolo[2,3-d]pyrimidine skeleton reach out of cavity and expose to solvent region. Therefore, we assume that there is a size limitation for cavity outlet of CDK9. Steric hindrance of 3,5-dimethylpyrimidin-2-amino group stopped small molecule into deeper spacious cavity to accommodate planar anisidine fragment. Removal of two methyl substituents on pyrimidine from **10a** could improve flexibility of small molecule within protein cavity. Thus, some CDK9 inhibitory activity was recovered in **10b** (54%). These four inactive compounds on CDK9 also lack of the activity against PDAC cell growth. This result, at least to some extent, confirmed the anticancer activity of this class of 2-((4-sulfamoylphenyl)amino)-pyrrolo[2,3-d]pyrimidine derivatives is related to CDK9 inhibition activity. Admittedly, the anticancer activity of these compounds is not strictly dependent on CDK9 inhibition from the data in Table 1. Therefore, several active compounds (IC_50_ < 10 µM) were further investigated in multiple CDK (1, 2, 4, 7, 9) assays. As a result, all these compounds were identified as CDK9 inhibitors but not CDK7 inhibitors. Meanwhile, they presented the differential effect on CDK1, 2, and 4. Comparing to **1**, compounds **5b**, **5c**, and **5e** exhibited higher selectivity preference for both CDK4 and 9. However, the most potent compound **2g** in PDAC cell culture behaves as a pan-CDK inhibitor which has strong inhibition on CDK1, 2, 4, and 9. Considering that ribociclib is a highly selective CDK4 inhibitor with low potency on CDK9, we estimated that inhibiting CDK9 enzyme activity played more important role to the anticancer performance of these derivatives in PDAC cells compared to other CDK subtypes.

#### Anti-proliferative activity screen in multiple PDAC cell lines

Thereafter, two potent anti-proliferative compounds **2g** and **5b**, which displayed different CDK inhibition profile, were investigated of anticancer activity in comparison with **1** in multiple human PDAC cell lines with different *Kras* genotypes. It could be seen that these compounds exerted higher sensitivity to BxPC-3 cells with wildtype *Kras* than *Kras*-mutant pancreatic cancer cell lines, which includes MIA PaCa-2, AsPC-1 and PANC-1. Moreover, two new derivatives **2g** and **5b** exhibited higher potency in some extend than compound **1** (Table 2). Surprisingly, all tested compounds showed significantly weaker potency in PANC-1 than other cell lines, and anti-proliferative activity of **1** was even slightly higher than both two new derivatives and gemcitabine in PANC-1 cell culture. To our knowledge, partial *Kras* G12D mutation was confirmed in PANC-1 while MIA PaCa-2 and AsPC-1 is fully *Kras* G12C and G12D mutation, respectively, by gene sequencing. We don’t know if *Kras* genetic status of cancer cells affects the biological performance of these CDK inhibitors in current study.

**Table 2.** Anti-proliferative activity of active 2-((4-sulfamoylphenyl)amino)-pyrrolo[2,3-d]pyrimidine derivatives in different PDAC cell lines.

Cpd.	IC_50_ (µM)^a^		
	AsPC-1	BxPC-3	PANC-1
**1**	2.598 ± 0.997	0.409 ± 0.060	3.679 ± 2.499
**2g**	1.403 ± 0.034	0.226 ± 0.065	11.022 ± 4.967
**5b**	1.479 ± 0.090	0.249 ± 0.099	5.814 ± 1.384
**GCTB**	0.956 ± 0.140	0.388 ± 0.119	5.789 ± 4.206

^a^Cell viability was determined after 72 h drug exposure to cells.

#### 2g induced cell cycle arrest and apoptosis in MIA PaCa-2 cells

**Figure 2.**
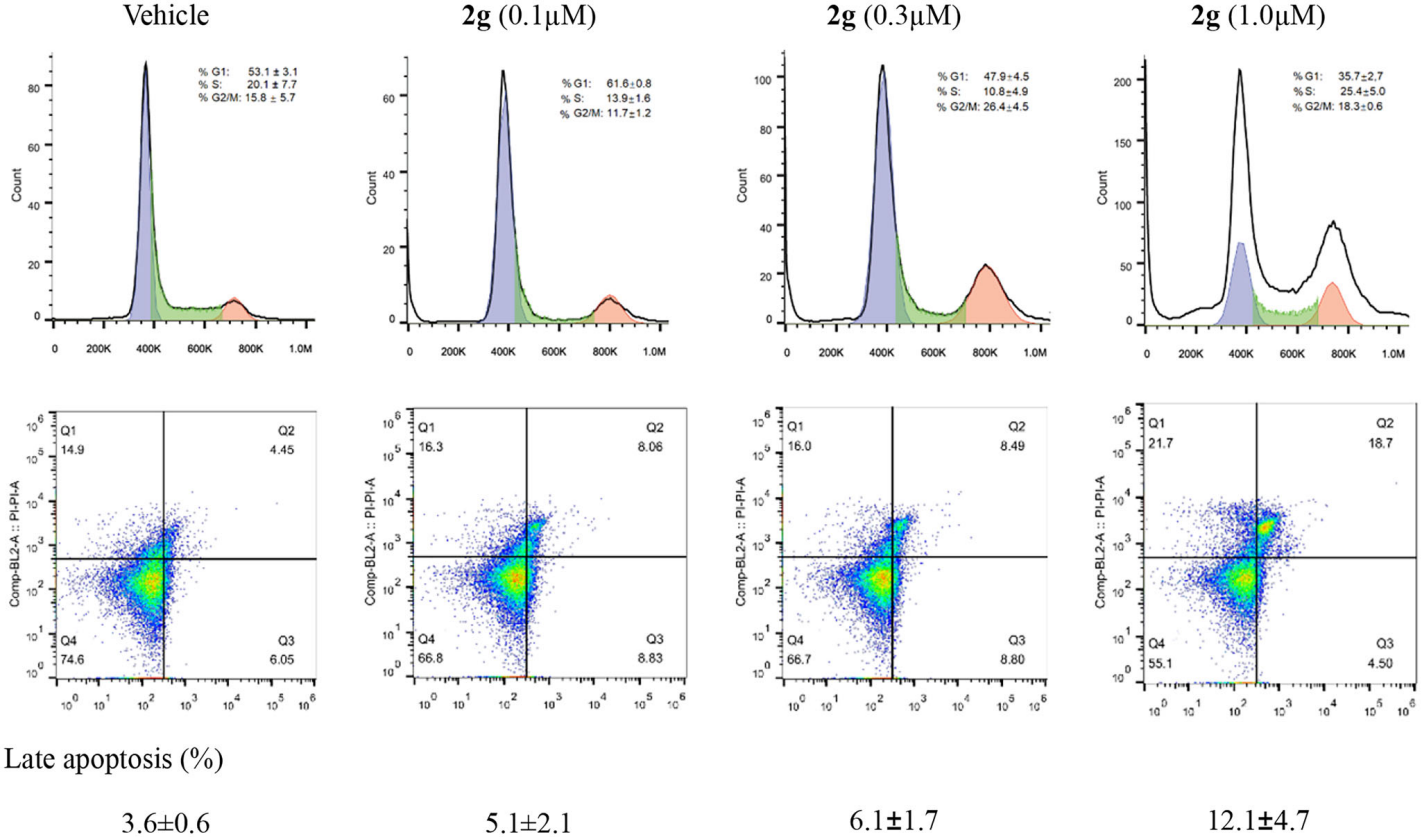
Compound **2g** induced slightly cell cycle arrest and late apoptosis of MIA PaCa-2 cells. The cells were incubated without or with **2g** at indicated concentrations for 24 h. The cell cycle distribution (Upper lane) or apoptosis (Lower lane) was detected using propidium iodide (PI) staining or both PI and annexin V-FITC staining, respectively, and analysed by flow cytometry assays. Graphs showing the results of a representative experiment, and the data was presented as mean ± standard deviation.

**Figure 3.**
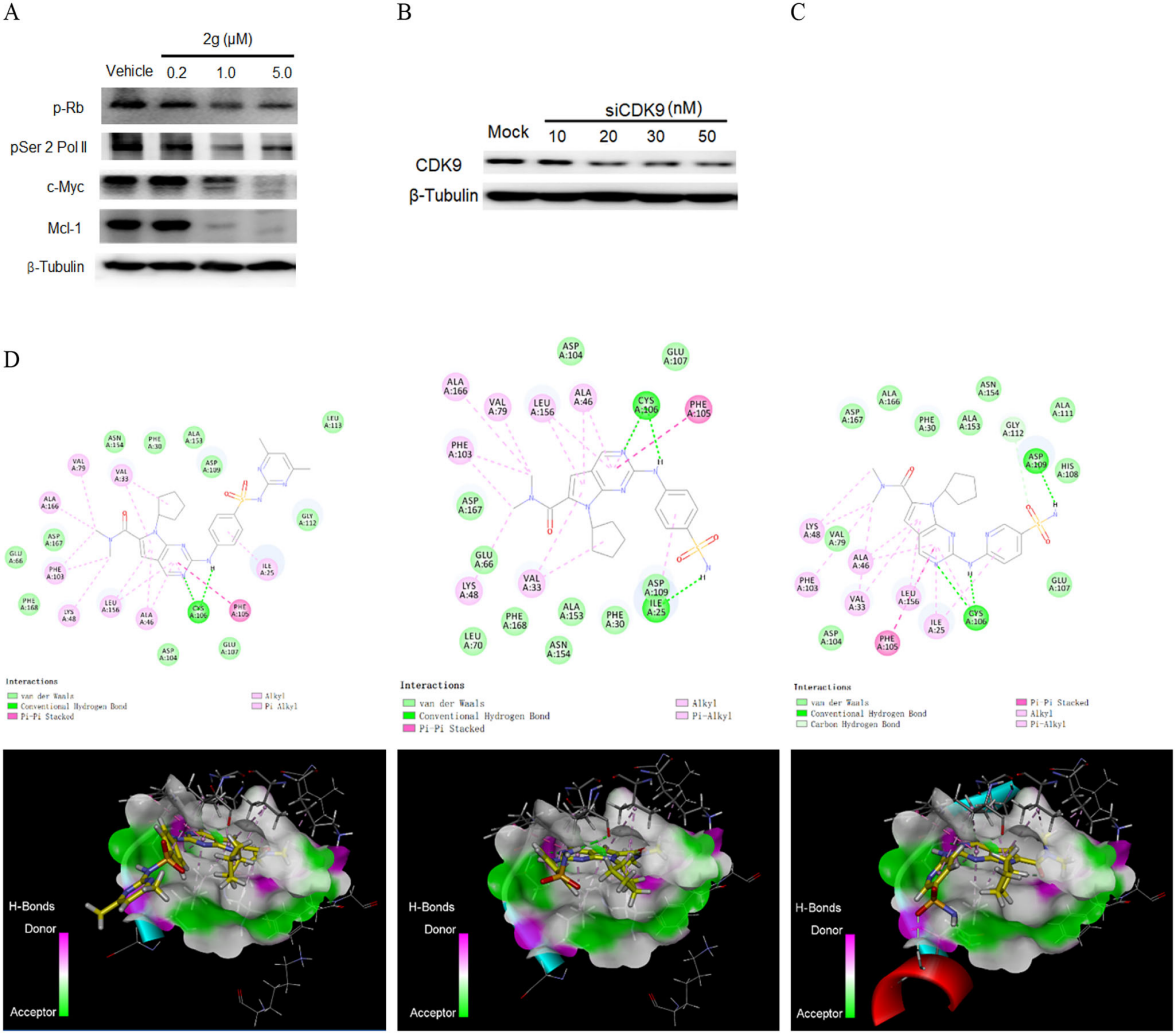
The anti-proliferative activity of new synthetic compounds is mainly mediated by CDK9. (A) **2g** inhibited the phosphorylation of Rb and RNA polymerase II, induced apoptosis through down-regulation of Mcl-1 and c-Myc protein. MIA PaCa-2 cells were incubated with or without **2g** at indicated concentration for 6 h. (B) CDK9 was dose-dependently knockdown by its specific siRNA after 48 h treatment in MIA PaCa-2 cells. (C) Downregulation of CDK9 using siCDK9 (50 nM) decreased the sensitivity of MIA PaCa-2 cell to compound **2g** (0.2 µM). The cells were incubated with **2g** for 72 h. NS no significance; *** *p* < 0.001; **** *p* < 0.0001. Whole-cell lysates were subjected to immunoblotting. A representative protein band of three independent experiments is shown. (D) Representative illustration of the binding of synthetic compound to CDK9/cyclin T. Small molecules **1** (left column), **2g** (middle column), and **2i** (right column) were shown in stick representation with carbons coloured yellow.

Based on above results, compound **2g** was further examined for the initial mechanism of action in MIA PaCa-2 cells. In previous study compound **1** displayed a G2-arrested effect on cell cycle of MIA PaCa-2 cells.^23^ Here, **2g** induced cell cycle arrest at G1 phase at low concentration of 0.1 µM after 24 h treatment of MIA PaCa-2 cells, which might be owing to the inhibition of **2g** on CDK4. As the concentration increased to 0.3 and 1.0 µM, a modest G2/M-arrested effect was observed (Figure 2, Upper). This result is consistent with the expected effect of CDK inhibitor. In apoptosis study (Figure 2, Lower), the number of late apoptotic cells increased in a dose-dependent manner after 24 h treatment with **2g**. It suggests that this compound inhibits the growth of MIA PaCa-2 cells by inducing apoptosis. Meanwhile, down-regulation of phosphorylated Rb protein was observed after **2g** treatment, this confirmed the effect of **2g** on cell cycle induced by regulation of Rb protein. In addition, inhibition of CDK9 could decrease the level of RNA polymerase II C-terminal domain serine 2 phosphorylation, the pro-survival protein Myeloid Cell Leukaemia 1 (Mcl-1) and MYC oncoprotein, and induced rapid apoptosis in cancer cells.^26^ In our study, same response in immunoblot assay was observed in MIA PaCa-2 cells after 6 h exposure to compound **2g** (Figure 3(A)). These results suggest that **2g** inhibited cancer cell proliferation by blocking Rb phosphorylation and induced apoptosis via downregulation of CDK9 downstream proteins. Compound **2g** displayed slightly weaker and significantly stronger inhibitory activity on CDK4 and CDK9, respectively, compared to CDK4 inhibitor ribociclib (Table 3). However, compound **2g** exhibited 20-fold higher *in vitro* anti-proliferative activity than the latter in MIA PaCa-2 cells (Table 1). Therefore, we thought that inhibition of CDK9 plays a key role in efficacy of compound **2g** in PDAC cell culture. To investigate the effect of CDK9 expression levels on PDAC cellular susceptibility to our synthetic compound, we used a specific siRNA of CDK9 to alter the protein expression in MIA PaCa-2 cells (Figure 3(B)). An obviously lower cell viability was observed when the cells was transfected with siRNA (50 nM) for 48 h compared to siRNA-untreated cells. Moreover, the sensitivity of MIA PaCa-2 cells to compound **2g** was significantly decreased when we treated cells with compound **2g** after downregulation of CDK9 by siRNA (Figure 3(C)). It suggests that the anti-proliferative activity of compound **2g** in MIA PaCa-2 cells relies on the inhibition of CDK9.

**Table 3.** IC_50_ (nM) values of anti-proliferative compounds against CDK activity.

Cpd.	IC_50_ (nM)^a^				
	CDK1	CDK2	CDK4	CDK7	CDK9
**1**	515.8	91.6	89	>10^4^	5.6
**2d**	ND^b^	7984.4	148	>10^4^	7.7
**2g**	54	21	12	>10^4^	5.0
**5b**	333	147	20	4967	3.2
**5c**	550	227	35	>10^4^	6.3
**5d**	906	2338.3	170	>10^4^	20
**5e**	439	235.9	52	>10^4^	13
**Ribociclib**	ND	ND	7.1	ND	2695
**Staurosporine**	2.5	0.9	28	70	15

^a^IC_50_ values of test compounds were determined with a serial of 3-fold diluted concentrations from top concentration of 10 μM and ATP concentration at Km in Mobility shift assay by single experiment.

^b^ND represents not determined.

### Molecular docking study

To better understand the interaction mode of synthetic compounds with CDK9, molecular docking study was carried out using the X-ray cocrystal of CDK9 complexed with a 4‑(thiazol-5-yl)-2-(phenylamino)pyrimidine CDK inhibitor (PDB Code: 4BCF)^27^. As shown in Figure 3(D), the N3-pyrrolo[2,3-d]pyrimidine of compound **1** accepts a hydrogen bond from the peptide nitrogen of Cys106 in hinge region, while the C2-NH of the pyrrolo[2,3-d]pyrimidine fused ring donates a hydrogen bond to the carbonyl of Cys106. Meanwhile, pyrrolo[2,3-d]pyrimidine is sandwiched between Ala46 and Leu156 by hydrophobic interaction, and pyrimidine ring contacts the gatekeeper residue (Phe105) to form a long π–π interaction at the distance of 5.38 Å. In addition, multiple van der Waals interactions were formed between compound **1** and hydrophobic residues within the active site of CDK9 complex. Owing to the relative malleability of the CDK9 active site, compound **2g** and other synthetic derivatives adopt a similar pose to compound **1** within the cavity of ATP binding site of CDK9 protein. In addition to above similar binding mode as compound **1**, the hydrogen of sulphonamide group of **2g** binds to Ile25 to form an additional hydrogen bond with CDK9. It was previously reported that replacing of aniline with 2-aminopyrimidine led to a clear preference to inhibition of CDK4/6 in the practical medicinal chemistry study. It was also observed from the comparison of CDK profiling between **2g** and **2i** in our research. However, we failed to explain the discrimination in CDK inhibitory activity through molecular docking analysis because both compounds exhibited similar interaction mode with CDK9/cyclinT complex (Figure 3(D), upper lane). The only difference of **2i** from **2g** in molecular docking study is that a smaller dihedral orientation between pyrrolo[2,3-d]pyrimidine fused ring and 2-aniline ring of compound **2i** was shown in 3 D diagram than that of the latter (Figure 3(D), lower lane). Therefore, we supposed that it brought two unfavourable effects to compound **2i**: one is that intramolecular exclusion due to excessive closing of 2-aminopyrimidine ring to 7-cyclopentyl ring; another is the space spatial location of N atom on the 2-aminopyrimidine ring is incompatible with the electrostatic environment of protein cavity in the practical experimental study. In summary, most of synthetic compounds kept the binding affinity to the active site of CDK9. The terminal of 2-aniline side chain of pyrrolo[2,3-d]pyrimidine scaffold extended to solvent exposure region. This explains why those active derivatives keep strong CDK9 inhibitory activity although they contain different substituents on sulphonyl group of 2-aniline moiety.

### *In vivo* pharmacokinetics (PK) study

**Figure 4.**
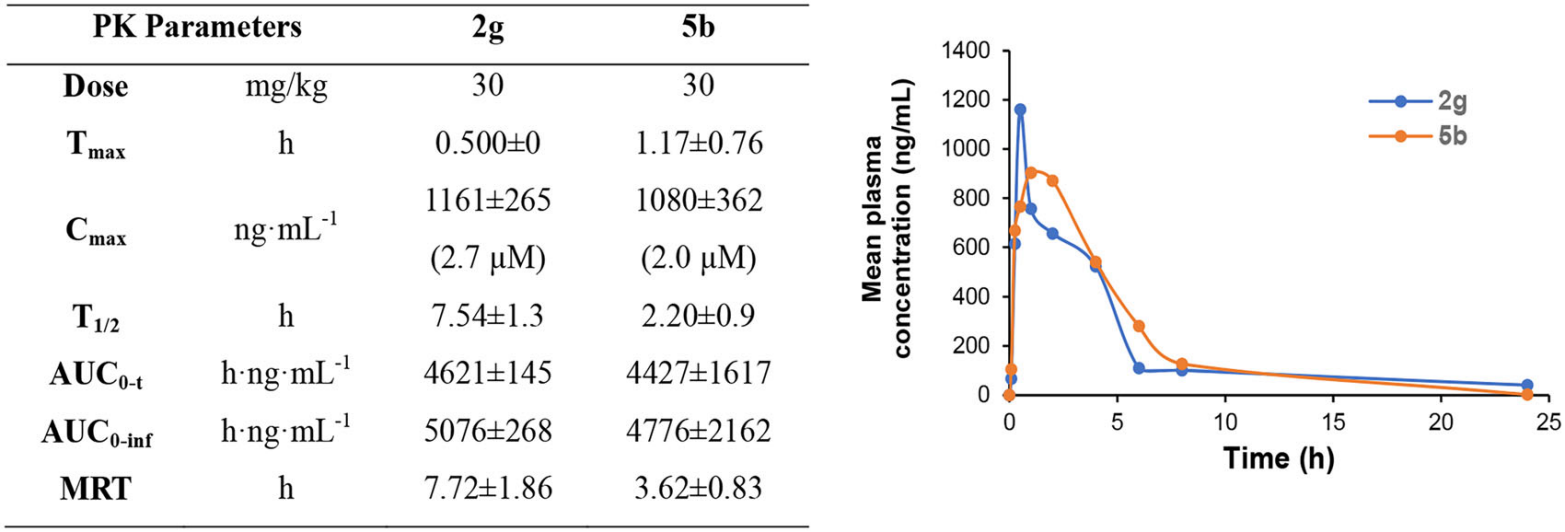
Oral PK study of compounds **2g** and **5 b** after oral administration to rats (*n* = 3) at dose of 30 mg/kg.

Although compounds **2g** and **5b** displayed different features in CDK selectivity, no significant difference of anticancer potency observed between them in multiple pancreatic cancer cell lines. So, oral absorption of both compounds was investigated in parallel by determining the drug concentration-time curve after single oral administration (Figure 4) to rats before the *in vivo* efficacy evaluation. Though solubility of **2g** was found to be much poorer than **5b** in the practical study, compound **2g** was absorbed into the circulation more rapidly (T_max_ = 0.5 h) and reached a slightly higher maximum plasma drug concentration (C_max_ = 1161 ng/mL·h) than that of **5b** (T_max_ = 1.17 h, C_max_ = 1080 ng/mL·h) after oral dosing to rats. Elimination half-life (T_1/2_) and mean retention time (MRT) of compound **2g** was calculated as 7.54 h and 7.72 h, respectively, which is significantly longer than **5b**. But we have to admit that rapid decrease of drug concentration to a low level within six hours occurred for both **2g** and **5b**. Finally, compound **2g** displayed a higher systematic exposure (AUC_0‑t_ = 4621 ng/mL·h) than **5b** (AUC_0‑t_ = 4427 ng/mL·h). Therefore, compound **2g** was selected to undergo *in vivo* efficacy study due to its slightly better performance in both oral PK features and antiproliferative activity than compound **5b** in this work.

### *In vivo* efficacy study

**Figure 5.**
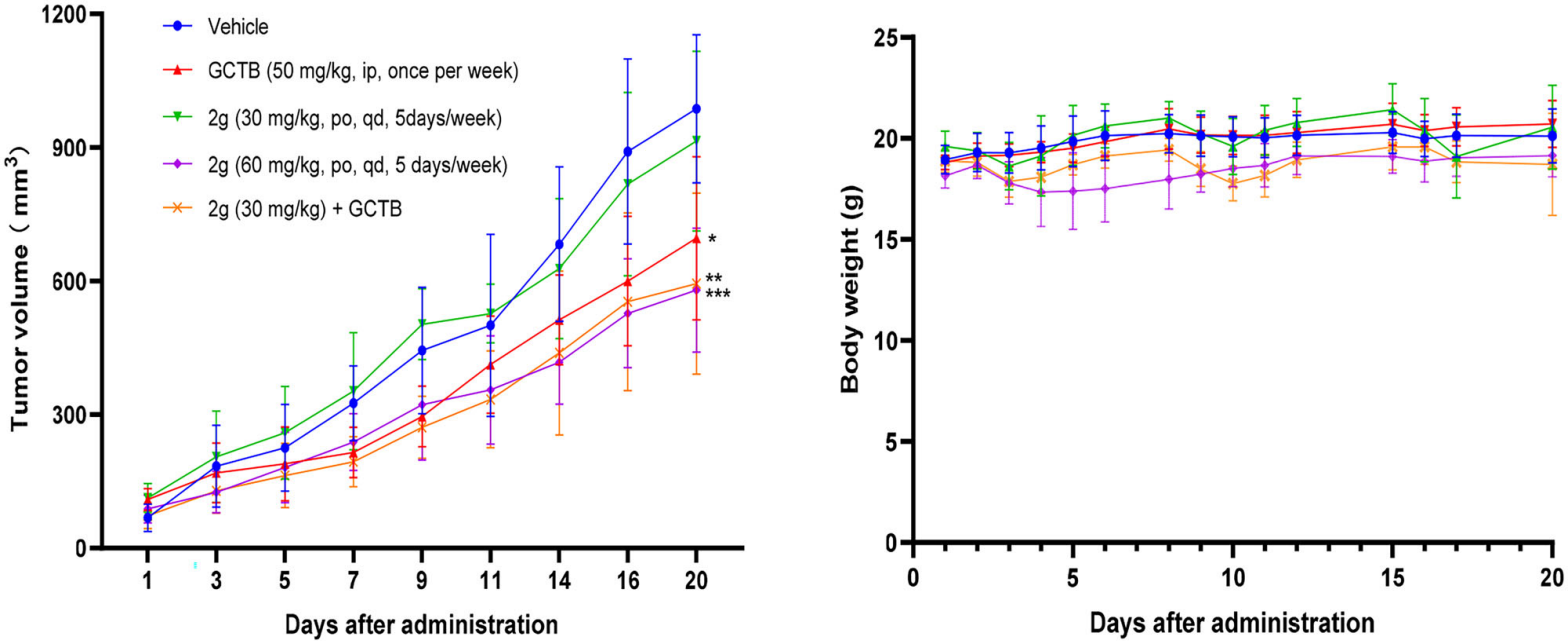
*In vivo* efficacy evaluation in AsPC-1 cell-derived xenograft mice model (*n* = 6).

**Figure 6.**
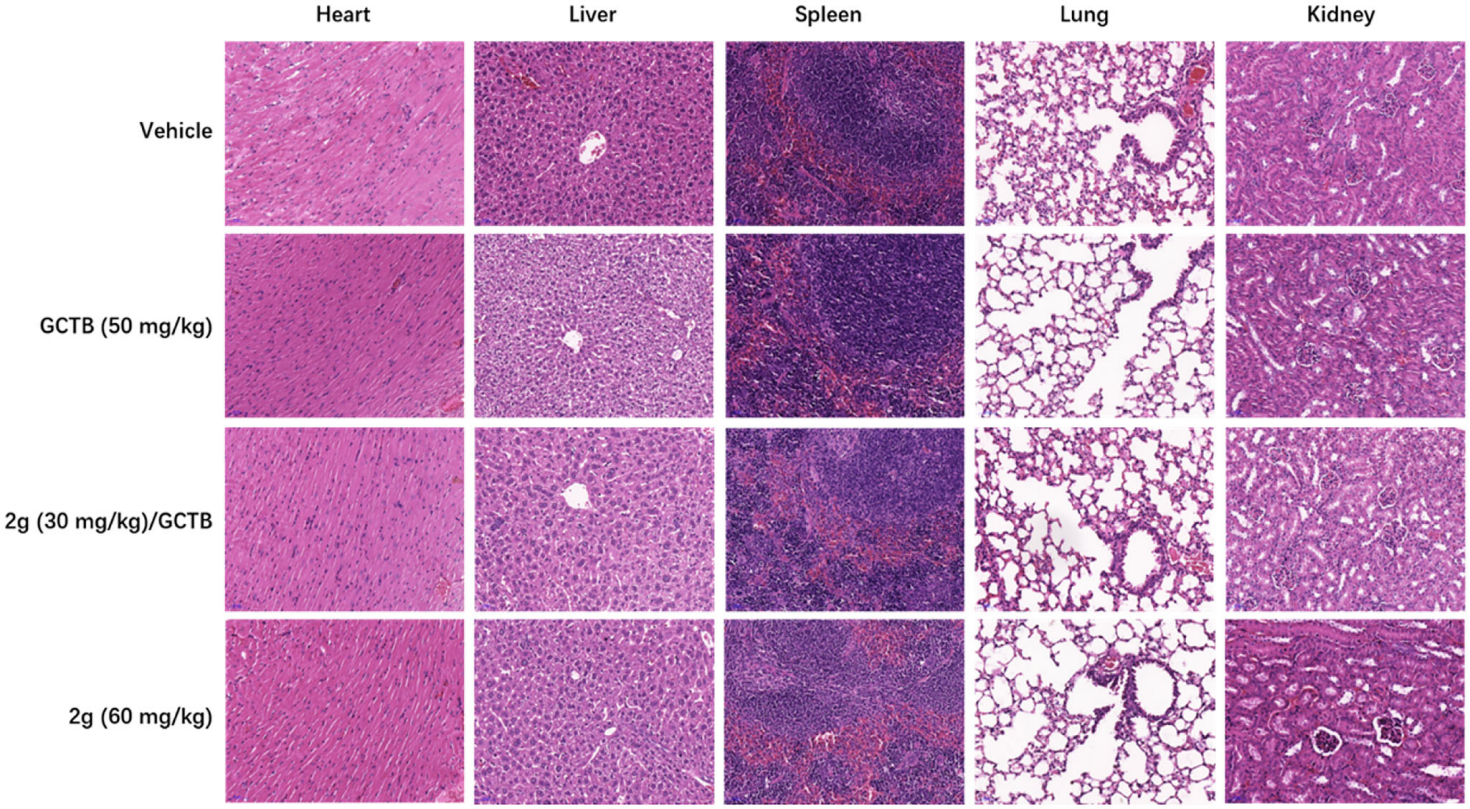
Representative H&E-stained tissue section (scale bar, 50 µM for all) from CDX mice treated with vehicle, GCTB or **2g** alone, or combination of GCTB and **2g** for 3 weeks.

To test if above cellular action could be translated into anti-pancreatic cancer activity *in vivo*, we used AsPC-1 line, which forms tumour more rapidly than MIA PaCa-2 in mice, to generate CDX subcutaneous xenograft mice model for evaluation of *in vivo* efficacy of **2g**. In addition of vehicle control, GCTB was used as a reference drug as it is the main chemotherapeutic agents used in clinic for pancreatic cancer. According to the PK data, we designed 30 mg/kg and 60 mg/kg oral dosages for **2g**. Drug treatment duration was three weeks with consecutive five days treatment and two days off per week. In addition, combining low dosage **2g** with GCTB was also included to explore the possibility of increasing GCTB susceptibility to pancreatic cancer in this work. As shown in Figure 5, GCTB exhibited tumour inhibition effect with tumour growth inhibition (TGI) value of 36% compared to vehicle group while **2g** at oral dose of 30 mg/kg only had a low TGI of 12% after 3-week administration. **2g** at dose of 60 mg/kg led to reduction of tumour volume up to 46% compared to vehicle group. Combination of GCTB with **2g** at dose of 30 mg/kg displayed higher TGI (43%) than either drug alone, but there is no significant difference between combination group and GCTB alone. Admittedly, an obvious decrease of body weight occurred in animals treated with high dose of **2g** during the first ten days of administration, and body weight subsequently came back to the normal level. The maximal individual body weight loss was 23% on Day 21 when **2g** was co-administered with GCTB. In addition, *in vivo* toxicity is a major concern due to the inhibitory activity of **2g** on multiple CDK kinase. Encouragingly, no obvious pathological change was observed in main organs of mice after 3-week treatment with either **2g** or GCTB (Figure 6).

To better understand *in vivo* efficacy of **2g**, we determined drug concentrations in both tumour tissues and plasma samples at 1 h and 4 h after 30 mg/kg single oral dosing to tumour-bearing mice. As shown in Table 4, drug concentration of **2g** in both plasma and tumour tissue is higher than 2-fold of cellular IC_50_ value. However, drug concentration significantly dropped down to an insufficient level for efficacy in either plasma and tumour tissue at 4 h after administration. This suggests that **2g** was easily omogenizat and eliminated, the resulted short target occupation might be the reason for its moderate *in vivo* efficacy.

**Table 4.** Drug concentrations in tumour tissue and plasma sample after 30 mg/kg single dosing of **2g** to tumour-bearing mice (*n* = 3).

Time after dosing (h)	Drug concentration (µM)	
	Tumour	Plasma
**1.0**	3.13 ± 0.64	7.46 ± 1.95
**4.0**	0.48 ± 0.34	0.53 ± 0.41

## Conclusions

A series of new 2-((4-sulfamoylphenyl)amino)-pyrrolo[2,3-d]pyrimidine derivatives was designed, omogenizat, and investigated for the inhibition effect on both human pancreatic cancer cell proliferation and CDK enzymatic activity. All anti-proliferative compounds in pancreatic MIA PaCa-2 cells displayed strong inhibition on CDK9 but no inhibition on CDK7. In addition, CDK4 activity might play a role in pancreatic cancer cell growth from the performance of active compounds in both *in vitro* anticancer activity and CDK inhibition pattern. Anti-proliferation MTT screening identified two highly active compounds **2g** and **5b** which kept a sensitivity at low micromolar level to both *Kras* -mutant and © PDAC cell lines. Especially, compound **2g** mediated cancer cell proliferation blockage and induced apoptosis in pancreatic cancer MIA PaCa-2 cells, and rapid down-regulation of Mcl-1 and c-Myc oncoprotein were observed after **2g** treatment. Moreover, CDK9 knockdown experiment suggests that the anti-proliferative activity of compound **2g** in MIA PaCa-2 cells is mainly mediated by the inhibition of CDK9. Initial molecular docking study revealed the principle binding mode of 2-((4-sulfamoylphenyl)amino)-pyrrolo[2,3-d]pyrimidine derivatives to CDK9/cyclin T complex. Detailed analysis might provide valuable cues for future design of new chemotype of selective CDK9 inhibitor. Oral PK and *in vivo* efficacy study further confirmed **2g** could inhibit tumour growth by oral administration for 3 weeks in AsPC-1 cell-derived xenograft mice model. Considering of function compensation of CDK7 and 9 in cancer cell proliferation, continuous effort to improve *in vivo* exposure of 2-((4-sulfamoylphenyl)amino)-pyrrolo[2,3-d]pyrimidine derivatives and screening effective anti-proliferative partners is worthy to provide promising regimens for PDAC treatment. Admittedly, CDK inhibition might not the only mechanism responsive for the anticancer activity of these 2-((4-sulfamoylphenyl)amino)-pyrrolo[2,3-d]pyrimidine derivatives. Benzenesulfonamide moiety appeared in the chemical structure of a variety of bioactive substances. For example, some benzenesulfonamide derivatives have been investigated and developed for the treatment of hypoxic tumours as carbonic anhydrase inhibitors^28^. Thus, deep study on anticancer mechanism of 2-((4-sulfamoylphenyl)amino)-pyrrolo[2,3-d]pyrimidine is necessary for further chemical omogenizatiation. Together, current study provided a new start to develop promising CDK inhibitor candidates for PDAC treatment by alone or combination use.

## Experimental methods

### General information for chemical experiments

All commercially available chemicals and solvents were used without further purification. The reaction was monitored by Merck TLC silica gel 60 F254 aluminium sheets or LC-MS instrument. Column chromatography was performed on RediSep silica gel column using the Teledyne ISCO CombiflashRf + system (Teledyne, Lincoln, USA) for purification. Samples purity were determined on a Shimadzu 2020 UFLC-MS instrument (Kyoto, Japan) which was equipped with a Shim-pack VP-ODS column (2.0 mm × 150 mm, 5 µm) and DAD detector, an electrospray omogenizaation source (ESI) and a single-quadrupole mass analyser. The elution phase was composed of acetonitrile and 0.1% formic acid aqueous solution in a gradient mode at a flow rate of 0.4 ml/min. The ^1^H spectrum were recorded using a WNMR-I 500 MHz spectrometer (Wuhan Zhongke-Niujin, China) or an INOVA 400 MHz spectrometer (Varian). HR-MS was collected with triple TOF 5600 + LC/MS/MS system (CADM-YQ-086) with an ESI mass selective detector (SCIEX Manufacturing, Singapore). All title compounds are >95% pure by UFLC analysis.

### Chemical synthesis

#### Preparation of 7-cyclopentyl-N,N-dimethyl-2-((4-(N-(4-methylpyrimidin-2-yl)sulfamoyl)phenyl)amino)-7H-pyrrolo[2,3-d]pyrimidine-6-carboxamide (2a)

To a solution of 2-chloro-7-cyclopentyl-N,N-dimethyl-7H-pyrrolo[2,3-d]pyrimidine-6-carboxamid-e (200 mg, 0.683 mmol) in DMF (10 ml) were added 4-amino-N-(4-methylpyrimidin-2-yl) benzenesulfonamide (163.94 mg, 1.35 mmol), XantPhos (107 mg, 0.186 mmol), Pd_2_(dba_3_) (113 mg, 0.124 mmol) and Cs_2_CO_3_ (606 mg, 186 mmol) at room temperature. The reaction was stirred at 100 °C until 13a was completely conversed into aim product by TLC monitoring. The mixture was filtered, concentrated and the residue was extracted with dichloromethane and distilled water. The water phase was reextracted with DCM for two times. The combined organic phase was washed with saline, dried with anhydrous magenism sulphate and filtered for purification by column chromatography eluted with dichloromethane/methamol (93/7) to afford the faint yellow solid **2a** with yield of 16.5%. ^1^H NMR (500 MHz, DMSO-d_6_) δ 10.04 (s, 1H), 8.81 (d, J = 1.6 Hz, 1H), 8.32 (d, J = 4.8 Hz, 1H), 7.97 (t, J = 10.5 Hz, 2H), 7.89 (d, J = 8.6 Hz, 2H), –.95–6.86 (m, 1H), –.65–6.60 (m, 1H), 5.75 (d, J = 1.5 Hz, 1H), 4.75 (q, J = 9.0 Hz, 1H), 3.06 (d, J = 8.7 Hz, 6H), 2.89 (d, J = 1.7 Hz, 1H), 2.73 (s, 1H), 2.44 (s, 2H), 2.32 (s, 3H), 1.98 (d, J = 17.8 Hz, 4H), 1.69 (s, 2H). HRMS(ESI+) m/z calcd C_25_H_29_N_8_O_3_S for [M + H] 521.2083, found 521.2069.

#### 7-Cyclopentyl-N,N-dimethyl-2-((4-(N-(pyrimidin-2-yl)sulfamoyl)phenyl)amino)-7H-pyrrolo[2,3-d]pyrimidine-6-carboxamide (2b)

It was omogenizatsised as yellow solid with yield of 31.8% from 2-chloro-7-cyclopenty-N,N-dimethyl-7H-pyrrolo[2,3-d]pyrimidine-6-carboxamide and 4-amino-N-(pyrimidin-2-yl)benzenesulfonamide by similar method as **2a**. ^1^H NMR (400 MHz, TFA-*d*) δ 8.99 (d, *J* = 5.5 Hz, 2H), 8.88 (s, 1H), –.33–8.26 (m, 2H), –.05–7.97 (m, 2H), 7.56 (t, *J* = 5.5 Hz, 1H), 6.97 (s, 1H), 4.75 (p, *J* = 8.9 Hz, 1H), –.41–3.23 (m, 6H), 2.50 (qd, *J* = 10.9, 8.7, 4.7 Hz, 2H), –.36–2.16 (m, 2H), –.14–2.00 (m, 2H), 1.84 (dq, *J* = 11.5, 7.1, 6.1 Hz, 2H). HRMS(ESI+) m/z calcd C_24_H_27_N_8_O_3_S for [M + H] 507.1927, found 507.1920.

#### 7-Cyclopentyl-N,N-dimethyl-2-((4omogeniridin-2-yl)sulfamoyl)phenyl)amino)-7H-pyrrolo[2,3-d]pyrimidine-6-carboxamide (2c)

It was omogenizatsised as yellow solid with yield of 24% from 2-chloro-7-cyclopenty-N,N-dimeth-yl-7H-pyrrolo[2,3-d]pyrimidine-6-carboxamide and 4-aminomogeniridin-2-yl)benzenesulfonamide by similar method as **2a**. ^1^H NMR (500 MHz, DMSO-d_6_) δ 10.01 (s, 1H), 8.83 (s, 1H), 8.09 (s, 1H), 7.98 (d, *J* = 8.5 Hz, 2H), 7.82 (d, *J* = 8.5 Hz, 2H), 7.72 (t, *J* = 7.6 Hz, 1H), 7.17 (d, *J* = 8.5 Hz, 1H), 6.92 (s, 1H), 6.65 (s, 1H), 4.78 (p, *J* = 8.8 Hz, 1H), 3.08 (s, 6H), 2.45 (s, 2H), –.08–1.91 (m, 4H), 1.70 (s, 2H). HRMS(ESI+) m/z calcd C_25_H_28_N_7_O_3_S for [M + H] 506.1974, found 506.1960.

#### 2-((4-(N-(4-chlorophenyl)sulfamoyl)phenyl)amino)-7-cyclopentyl-N,N-dimethyl-7H-pyrrolo[2,3-d]pyrimidine-6-carboxamide (2d)

It was omogenizatsised as white solid with yield of 28.3% from 2-chloro-7-cyclopenty-N,N-dimethyl-7H-pyrrolo[2,3-d]pyrimidine-6-carboxamide and 4-amino-N-(4-chlorophenyl)benzenesulfonamide by similar method as **2a**. ^1^H NMR (500 MHz, DMSO-d_6_) δ 10.14 (dd, J = 97.2, 5.9 Hz, 2H), 8.81 (d, J = 6.5 Hz, 1H), 7.93 (q, J = 5.7, 3.7 Hz, 2H), –.74–7.52 (m, 2H), 7.28 (d, J = 7.1 Hz, 2H), –.19–7.03 (m, 2H), 6.63 (d, J = 6.3 Hz, 1H), –.82–4.65 (m, 1H), 3.05 (s, 6H)2.38 (d, J = 14.1 Hz, 2H), 1.94 (d, J = 43.3 Hz, 4H), 1.65 (s, 2H). HRMS(ESI+) m/z calcd C_26_H_28_ClN_6_O_3_S for [M + H] 539.1632, found 539.1634.

#### 7-Cyclopentyl-2-((4-(N,N-dimethylsulfamoyl)phenyl)amino)-N,N-dimethyl-7H-pyrrolo[2,3-d]pyrimidine-6-carboxamide (2e)

It was omogenizatsised as white solid with yield of 49.5% from 2-chloro-7-cyclopenty-N,N-dimethyl-7H-pyrrolo[2,3-d]pyrimidine-6-carboxamide and 4-amino-N,N-dimethylbenzenesulfonamide by similar method as **2a**. ^1^H NMR (500 MHz, Chloroform-*d*) δ 8.68 (s, 1H), 7.93 (s, 1H), 7.89 (d, J = 8.3 Hz, 2H), 7.77 − 7.70 (m, 2H), 6.48 (s, 1H), 4.80 (p, J = 9.0 Hz, 1H), 3.16 (s, 6H), 2.72 (s, 6H), –.59–2.51 (m, 2H), –.14–2.03 (m, 4H), –.78–1.69 (m, 2H). HRMS(ESI+) m/z calcd C_22_H_29_N_6_O_3_S for [M + H] 457.2022, found 457.2025.

#### 7-Cyclopentyl-N,N-dimethyl-2-((4-(methylsulfonyl)phenyl)amino)-7H-pyrrolo[2,3-d]pyrimidine-6-carboxamide (2f)

It was omogenizatsised as white solid with yield of 31.9% from 2-chloro-7-cyclopenty-N,N-dimethyl-7H-pyrrolo[2,3-d]pyrimidine-6-carboxamide and 4-(methylsulfonyl)aniline by similar method as **2a**. ^1^H NMR (500 MHz, Chloroform-*d*) δ 8.69 (s, 1H), –.95–7.84 (m, 5H), 6.47 (s, 1H), –.87–4.74 (m, 1H), 3.16 (s, 6H), 3.06 (s, 3H), –.64–2.51 (m, 2H), 2.08 (dd, J = 17.5, 7.6 Hz, 4H), –.79–1.70 (m, 2H). HRMS(ESI+) m/z calcd C_21_H_26_N_5_O_3_S for [M + H] 428.1751, found 428.1761.

#### 7-Cyclopentyl-N,N-dimethyl-2-((4-sulfamoylphenyl)amino)-7H-pyrrolo[2,3-d]pyrimidine-6-carboxamide (2 g)

It was omogenizatsised as white solid with yield of 22.9% from 2-chloro-7-cyclopenty-N,N-dimethyl-7H-pyrrolo[2,3-d]pyrimidine-6-carboxamide and 4-aminobenzenesulfonamide by similar method as **2a**. ^1^H NMR (600 MHz, DMSO-d_6_) δ 9.96 (s, 1H), 8.82 (s, 1H), 8.00 (d, J = 8.9 Hz, 2H), 7.73 (d, J = 8.9 Hz, 2H), 7.15 (s, 2H), 6.63 (s, 1H), 4.76 (p, J = 8.8 Hz, 1H), 3.06 (s, 6H), 2.46 (dd, J = 14.7, 6.0 Hz, 2H), –.08–1.94 (m, 4H), –.74–1.64 (m, 2H). HRMS(ESI+) m/z calcd C_20_H_25_N_6_O_3_S for [M + H] 429.1709, found 429.1692.

#### 7-Cyclopentyl-N,N-dimethyl-2-((5-sulfamoylpyridin-2-yl)amino)-7H-pyrrolo[2,3-d]pyrimidine-6-carboxamide (2i)

It was omogenizatsised as white solid with yield of 29.8% from 2-chloro-7-cyclopenty-N,N-dimethyl-7H-pyrrolo[2,3-d]pyrimidine-6-carboxamide and 6-aminopyridine-3-sulphonamide by similar method as **2a**. ^1^H NMR (500 MHz, DMSO-d_6_) δ 10.44 (s, 1H), 8.91 (s, 1H), 8.68 (d, J = 2.5 Hz, 1H), 8.49 (d, J = 9.0 Hz, 1H), 8.12 (dd, J = 9.0, 2.6 Hz, 1H), 7.41 (s, 2H), 6.69 (s, 1H), 4.78 (p, J = 8.8 Hz, 1H), 3.07 (d, J = 8.2 Hz, 6H), –.48–2.39 (m, 2H), –.07–1.95 (m, 4H), 1.70 (dd, J = 11.5, 5.4 Hz, 2H).HRMS(ESI+) m/z calcd C_19_H_24_N_7_O_3_S for [M + H] 430.1661, found 430.1661.

#### 7-Cyclopentyl-2-((5-(N,N-dimethylsulfaomogeniridin-2-yl)amino)-N,N-dimethyl-7H-pyrrolo[2,3-d]pyrimidine-6-carboxamide (2j)

It was omogenizatsised as white solid with yield of 31.6% from 2-chloro-7-cyclopenty-N,N-dimethyl-7H-pyrrolo[2,3-d]pyrimidine-6-carboxamide and 6-amino-N,N-dimethylpyridine-3-sulphonamide by similar method as **2a**. ^1^H NMR (500 MHz, DMSO-d_6_) δ 10.51 (s, 1H), 8.93 (s, 1H), 8.62 (d, J = 2.5 Hz, 1H), 8.51 (d, J = 9.0 Hz, 1H), 8.09 (dd, J = 9.0, 2.5 Hz, 1H), 6.71 (s, 1H), 4.82 (p, J = 8.9 Hz, 1H), 3.09 (s, 6H), 2.68 (s, 6H), 2.42 (dd, J = 14.8, 6.7 Hz, 2H), 2.03 (q, J = 9.9, 8.7 Hz, 4H), –.73–1.64 (m, 2H). HRMS(ESI+) m/z calcd C_21_H_28_N_7_O_3_S for [M + H] 458.1974, found 458.1958.

#### Preparation of tert-Butyl 4-((4-nitrophenyl)sulphonyl)piperazine-1-carboxylate (3a)

To a solution of tert-butyl piperazine-1-carboxylate (252 mg, calculated as 1.35 mmol) and K_2_CO_3_ (373 mg, 2.7 mmol) in acetonitrile (10 ml) p-nitrobenzenesulfonyl chloride (300 mg, 1.35 mmol) was added slowly, and the reaction was started at room temperature. The reaction was completed as shown in TLC plate. The mixture was filtered, concentrated and the residue was extracted with dichloromethane and distilled water. The organic phase was washed with saline, concentrated and dried to afford **3a** as yellow solid by yield of 73%. ^1^H NMR (400 MHz, DMSO-d_6_) δ 8.43–8.36(m, 2H), 8.00–7.91 (m, 2H), 3.35 (t, = 5.1 Hz, 4H), 2.90 (t, *J* = 5.1 Hz, 4H), 1.29 (s, 9H).

#### 1-Methyl-4-((4-nitrophenyl)sulphonyl)piperazine (3b)

It was omogenizatsised as faint red solid with yield of 83.6% from *p*-nitrobenzenesulfonyl chloride and 1-methylpiperazine by similar method as **3a**. ^1^H NMR (400 MHz, DMSO-d_6_) δ 8.47–8.42(m, 2H), 8.03–7.98 (m, 2H), 2.97 (t, *J* = 5.0 Hz, 4H), 2.37 (t, *J* = 4.9 Hz, 4H), 2.15 (s, 3H).

#### 4-Methyl-1-((4-nitrophenyl)sulphonyl)piperidine (3c)

It was omogenizatsised as white-like solid solid with yield of 87% from p-nitrobenzenesulfonyl chloride and 4-methylpiperidine by similar method as **3a**. ^1^H NMR(500 MHz,Chloroform-*d*) δ 8.40(d, *J* = 8.4 Hz,2H),7.97(d, *J* = 8.3 Hz,2H), 3.82 (d, *J* = 11.0 Hz, 2H),2.35(t,*J* = 11.3 Hz,2H),1.72(d, *J* = 10.4 Hz, 2H),1.41–1.31 (m, 2H), 1 0.29 (dd, *J* = 11.3, 3.9 Hz, 1H), 0.95 (d, *J* = 5.3 Hz, 3H).

#### 4-((4-Nitrophenyl)sulphonyl)morpholine (3d)

It was omogenizatsised as white-like solid solid with yield of 83% from p-nitrobenzenesulfonyl chloride and morpholine by similar method as **3a**. ^1^H NMR (500 MHz, Chloroform-*d*) δ 8.49–8.40(m,2H), 7.99 (dd, J = 8.9, 2.1 Hz, 2H), 3.80 (t, *J* = 4.7 Hz, 4H), 3.10 (dd, *J* = 5.8,3.5 Hz, 4H).

#### Preparation of tert-Butyl 4-((4-aminophenyl)sulphonyl)piperazine-1-carboxylate (4a)

To a solution of **3a** (500 mg, 1.75 mmol) in DCM (20 ml) Pd/C (50 mg) was added. The reaction was stirred for 3 h at 30 psi. The reaction was completed as shown in TLC plate. The mixture was filtered and the filtrate was concentrated and dried to afford **4a** as white solid by yield of 96.1%. ^1^H NMR (400 MHz, Chloroform-*d*) δ 7.59–7.53(m, 2H), 6.77–6.70 (m, 2H), 4.20 (s, 2H), 3.57–3.51(m, 4H), 2.97 (t, *J* = 5.0 Hz, 4H), 1.45 (s, 10H).

#### 4-((4-Methylpiperazin-1-yl)sulphonyl)aniline (4b)

It was omogenizatsised as white solid solid with yield of 41.7% from **3b** and Pd/C by similar method as **4a**. ^1^H NMR (400 MHz, DMSO-d_6_) δ 7.37–7.31 (m, 2H), 6.67–6.61 (m, 2H), 6.07 (s, 2H), 2.79 (s, 4H), 2.35 (s, 4H), 2.14 (s, 3H).

#### 4-((4-Methylpiperidin-1-yl)sulphonyl)aniline (4c)

It was omogenizatsised as white solid solid with yield of 97.4% from **3c** and Pd/C by similar method as **4a**. ^1^H NMR (400 MHz, Chloroform-*d*) δ 7.56 (d, *J* = 8.3 Hz, 2H), 6.73 (d, *J* = 8.3 Hz, 2H), 4.15 (s, 2H), 3.73 (d, *J* = 10.9 Hz, 2H), 2.33–2.11 (m, 2H), 1.69 (d, *J* = 9.5 Hz, 2H), 1.33 (d, *J* = 10.8 Hz, 3H), 0.94 (d, *J* = 4.4 Hz, 3H).

#### 4-(Morpholinosulfonyl)aniline (4d)

It was omogenizatsised as white solid solid with yield of 91.9% from **3d** and Pd/C by similar method as **4a**. ^1^H NMR (400 MHz, Chloroform-*d*) δ 7.58–7.50 (m, 2H), 6.74–6.64(m, 2H), 4.15 (s, 2H), 3.14 (t, *J* = 4.7 Hz, 4H), 2.96 (t, *J* = 4.7 Hz, 4H).

#### Tert-Butyl 4-((4-((7-cyclopentyl-6-(dimethylcarbamoyl)-7H-pyrrolo[2,3-d]pyrimidin-2-yl)amino)phenyl)sulphonyl)piperazine-1-carboxylate (5a)

It was omogenizatsised from 2-chloro-7-cyclopenty-N,N-dimethyl-7H-pyrrolo[2,3-d]pyrimidine-6-carboxamide and **4a** by similar method to **2a** as white-like solid (yield: 71%). ^1^H NMR (500 MHz, DMSO-d_6_) δ 10.12 (s, 1H), 8.85 (s, 1H), 8.07 (d, J = 8.5 Hz, 2H), 7.65 (d, J = 8.4 Hz, 2H), 6.66 (s, 1H), 4.80 (s, 1H), 3.42 (s, 4H), 3.08 (s, 6H), 2.85 (s, 4H), 2.44 (s, 2H), 2.00 (d, J = 9.4 Hz, 4H), 1.69 (s, 2H), 1.35 (s, 9H).

#### Preparation of 7-Cyclopentyl-N,N-dimethyl-2-((4-(piperazin-1-ylsulfonyl)phenyl)amino)-7H-pyrrolo[2,3-d]pyrimidine-6-carboxamide trifluoroacetate (5b)

To a solution of **5a** (1.53 g) in the dichloromethane (15 ml) trifluoroacetic acid (2.0 ml) was added. The reaction was heated at 55 °C and stirred until **5a** was absence by LC-MS. The mixture was concentrated to remove the solvent and resuspended in the ether to afford white-like powder **5b** in the yield of 90%. ^1^H NMR (500 MHz, DMSO-d_6_) δ 10.29 (s, 1H), 9.13 (s, 1H), 8.89 (s, 1H), 8.13 (d, J = 8.7 Hz, 2H), 7.71 (d, J = 8.7 Hz, 2H), 6.70 (s, 1H), 4.81 (dd, J = 17.4, 8.8 Hz, 1H), 3.18 (d, J = 14.7 Hz, 8H), 3.06 (d, J = 24.0 Hz, 6H), 2.42 (d, J = 29.8 Hz, 2H), –.10–1.94 (m, 4H), 1.71 (d, J = 5.0 Hz, 2H). HRMS(ESI+) m/z calcd C_24_H_32_N_7_O_3_S for [M + H] 498.2287, found 498.2274.

#### 7-Cyclopentyl-N,N-dimethyl-2-((4-((4-methylpiperazin-1-yl)sulphonyl)phenyl)amino)-7H-pyrrolo[2,3-d]pyrimidine-6-carboxamide (5c)

It was omogenizatsised as white solid with yield of 33% from 2-chloro-7-cyclopenty-N,N-dimethyl-7H-pyrrolo[2,3-d]pyrimidine-6-carboxamide and **4b** by similar method as **2a**. ^1^H NMR (600 MHz, DMSO-d_6_) δ 10.08 (s, 1H), 8.84 (s, 1H), 8.06 (t, J = 5.7 Hz, 2H), 7.63 (d, J = 8.9 Hz, 2H), 6.64 (s, 1H), 4.78 (p, J = 8.9 Hz, 1H), 3.06 (s, 6H), 2.88 (s, 4H), 2.44 (dd, J = 19.9, 8.1 Hz, 2H), 2.38 (d, J = 17.1 Hz, 4H), 2.14 (s, 3H), –.05–1.92 (m, 4H), –.72–1.63 (m, 2H). HRMS(ESI+) m/z calcd C_25_H_34_N_7_O_3_S for [M + H] 512.2444, found 512.2436.

#### 7-Cyclopentyl-N,N-dimethyl-2-((4-((4-methylpiperidin-1-yl)sulphonyl)phenyl)amino)-7H-pyrrolo[2,3-d]pyrimidine-6-carboxamide (5c)

It was omogenizatsised as white solid with yield of 61% from 2-chloro-7-cyclopenty-N,N-dimethyl-7H-pyrrolo[2,3-d]pyrimidine-6-carboxamide and **4c** by similar method as **2a**. ^1^H NMR (500 MHz, DMSO-d_6_) δ 10.07 (s, 1H), 8.83 (d, J = 1.9 Hz, 1H), 8.03 (d, J = 8.5 Hz, 2H), 7.62 (d, J = 8.5 Hz, 2H), 6.64 (d, J = 1.8 Hz, 1H), 4.78 (p, J = 8.8 Hz, 1H), 3.58 (d, J = 11.3 Hz, 2H), 3.06 (d, J = 10.1 Hz, 6H), 2.42 (t, J = 9.9 Hz, 2H), 2.17 (t, J = 11.9 Hz, 2H), 1.98 (dt, J = 15.8, 9.1 Hz, 4H), 1.65 (t, J = 11.8 Hz, 4H), 1.28 (s, 1H), –.19–1.05 (m, 2H), 0.84 (d, J = 6.3 Hz, 3H). HRMS(ESI+) m/z calcd C_26_H_35_N_6_O_3_S for [M + H] 511.2491, found 511.2468.

#### 7-Cyclopentyl-N,N-dimethyl-2-((4-(morpholinosulfonyl)phenyl)amino)-7H-pyrrolo[2,3-d]pyrimidine-6-carboxamide (5d)

It was omogenizatsised as white solid with yield of 31.9% from 2-chloro-7-cyclopenty-N,N-dimethyl-7H-pyrrolo[2,3-d]pyrimidine-6-carboxamide and **4d** by similar method as **2a**. ^1^H NMR (500 MHz, DMSO-d_6_) δ 10.12 (s, 1H), 8.84 (d, J = 1.9 Hz, 1H), 8.07 (d, J = 8.5 Hz, 2H), 7.63 (d, J = 8.5 Hz, 2H), 6.64 (d, J = 2.0 Hz, 1H), 4.78 (p, J = 8.9 Hz, 1H), 3.63 (d, J = 9.3 Hz, 4H), –.12–2.99 (m, 6H), 2.85 (t, J = 4.7 Hz, 4H), 2.43 (q, J = 9.9, 9.1 Hz, 2H), 2.00 (dq, J = 24.6, 8.2, 7.7 Hz, 4H), 1.67 (q, J = 6.7, 6.0 Hz, 2H). HRMS(ESI+) m/z calcd C_24_H_31_N_6_O_4_S for [M + H] 499.2127, found 499.2113.

#### Preparation of 2-(cyclopentylamino)-N-(2-methoxyphenyl)acetamide (6)

As previously described,^23^ to a solution of 2-methoxyphenylamine in acetonitrile, diisopropylethylamine (1.0 equiv) and 2-chloroacetyl chloride (1.0 equiv) were slowly added under ice-salt bath. After 0.5 h, cyclopentylamine (4.0equiv) was added into above mixture and the reaction was stirred at room temperature overnight. The resultant reaction was filtered, and concentrated *under vacuo* to remove the solvent and excessive cyclopentylamine. The residue was extracted with ethyl acetate and water, organic phase was washed with saline, dried with anhydrous sodium sulphate and concentrated *under vacuo* to afford crude **6** as a dark yellow oil (56%). ^1^H NMR (500 MHz, DMSO-d_6_) δ 10.01 (s,1H), 8.76 (s, 1H), 8.29 (d, *J* = 4.1 Hz, 1H), 8.09 (d, *J* = 8.0 Hz, 1H), 7.36 (dt, *J* = 27.1, 13.5 Hz, 1H), 3.41–3.38 (s,3H), –.15–2.97 (m, 1H), –.81–1.59 (m, 4H), –.58–1.26 (m, 4H).

#### One-pot method for synthesis of 7-cyclopentyl-N-(2-methoxyphenyl)-2-(methylthio)-7H-pyrrolo[2,3-d]pyrimidine-6-carboxamide (8)

To solution of **6** (1.53 g, 6.0 mmol) and DIPEA (1.04 g, 8.36 mmol) in acetonitrile (20 ml), commercially available 4-chloro-2-(methylthio)pyrimidine-5-carbaldehyde (1.30 g, 6.9 mmol) was added slowly at room temperature. The reaction was stirred for 3 h at 50 °C to afford intermediate **7**. Caesium carbonate (5.6 g, 17.28 mmol) was added into above reaction and stirred under MW irradiation for 0.5 h at 120 °C. LC-MS showed that the reaction was completed. The resulted mixture was filtered, evaporated and extracted with ethyl acetate and water. The organic phase was dried and evaporated to remove the solvent. The residue was recrystallized with methanol to afforded **8** as white solid (1.9 g) in the yield of 68%. ^1^H NMR (500 MHz, DMSO-d_6_) δ 9.68 (s, 1H), 8.99 (s, 1H), 7.80 (d, *J* = 7.7 Hz, 1H), 7.23 (t, *J* = 7.4 Hz, 2H), 7.13 (d, *J* = 8.1 Hz, 1H), 7.00 (t, *J* = 7.6 Hz, 1H), 5.52 (p, *J* = 8.6 Hz, 1H), 3.87 (s, 3H), 2.60 (s, 3H), –.50–2.38 (m, 2H), 2.04 (dd, *J* = 12.8, 6.7 Hz, 4H), 1.67 (d, *J* = 5.4 Hz, 2H).

#### 7-Cyclopentyl-N-(2-methoxyphenyl)-2-(methylsulfonyl)-7H-pyrrolo[2,3-d]pyrimidine-6-carboxamide (9)

To a solution of **8** (1 equiv) in DCM (20 ml), m-CPBA (3 equiv) was slowly added and the reaction was heated under reflux for 2 – 3 h, then cooled to room temperature and evaporated *under vacuo*. The residue was extracted with ethyl acetate and saturated aqueous sodium bicarbonate, and washed with brine. The organic phase was dried with sodium sulphate and evaporated to remove the solvent. The residue was purified by flash column chromatography to give the desired product as light pink solid with yield of 57%. ^1^H NMR (500 MHz, Chloroform-*d*) δ 9.22 (s, 1H), 8.62 (s, 1H), 8.50 (d, *J* = 7.7 Hz, 1H), 7.21 (t, *J* = 7.8 Hz, 1H), –.13–7.07 (m, 2H), 7.02 (d, *J* = 8.1 Hz, 1H), 5.65 (dd, *J* = 17.3, 8.6 Hz, 1H), 4.00 (s, 3H), 3.46 (s, 3H), 2.51 (d, *J* = 7.8 Hz, 2H), –.31–2.11 (m, 4H), 1.79 (d, *J* = 4.8 Hz, 2H).

#### 7-Cyclopentyl-2-((4-(N-(4,6-dimethylpyrimidin-2-yl)sulfamoyl)phenyl)amino)-N-(2-methoxyphenyl)-7H-pyrrolo[2,3-d]pyrimidine-6-carboxamide (10a)

To a solution of **9** (150 mg) in DMF (5 ml), N-(4-(N-(4,6-dimethylpyrimidin-2-yl)sulfamoyl)phenyl)formamide (110 mg) and caesium carbonate (235 mg) were added. The reaction was heated at 85 °C for 24 h. Then the mixture was extracted with ethyl acetate and the distilled water, the organic phase was washed with brine, concentrated under vacuo, and suspended in the methanol. The product **10a** was obtained after filtration as white-like solid. (96 mg, 43%). ^1^H NMR (400 MHz, DMSO-d_6_) δ 10.13 (s, 1H), 9.54 (s, 1H), 8.93 (s, 1H), 8.01 (d, *J* = 8.8 Hz, 2H), 7.92 (d, *J* = 8.8 Hz, 2H), 7.79 (dd, *J* = 7.9, 1.6 Hz, 1H), –.26–7.16 (m, 2H), 7.11 (dd, *J* = 8.4, 1.4 Hz, 1H), 6.98 (td, *J* = 7.6, 1.4 Hz, 1H), 6.78 (s, 1H), 5.51 (p, *J* = 9.2 Hz, 1H), 3.85 (s, 3H), 2.55 (s, 3H), 2.26 (s, 7H), 2.01 (s, 5H), 1.71 (s, 2H). HRMS(ESI+) m/z calcd C_31_H_33_N_8_O_4_S for [M + H] 613.2349, found 613.2349.

#### 7-Cyclopentyl-N-(2-methoxyphenyl)-2-((4-(N-(pyrimidin-2-yl)sulfamoyl)phenyl)amino)-7H-pyrrolo[2,3-d]pyrimidine-6-carboxamide (10b)

It was prepared by the reaction of **9** with N-(4-(N-(pyrimidin-2-yl)sulfamoyl)phenyl)formamide using the same method as **10a** in the yield of 38% as a white-like solid. ^1^H NMR (400 MHz, TFA-*d1*) δ 9.02 (t, *J* = 7.9 Hz, 2H), 8.96 (s, 1H), 8.34 (d, *J* = 8.4 Hz, 2H), 8.05 (t, *J* = 7.8 Hz, 3H), 7.61 (t, *J* = 5.3 Hz, 1H), –.42–7.34 (m, 2H), 7.13 (t, *J* = 8.2 Hz, 2H), 5.40 (p, *J* = 8.9 Hz, 1H), 4.02 (s, 3H), 2.58 (d, *J* = 7.9 Hz, 2H), 2.33 (d, *J* = 20.3 Hz, 2H), 2.11 (s, 2H), 1.87 (d, *J* = 5.1 Hz, 2H). HRMS(ESI+) m/z calcd C_29_H_29_N_8_O_4_S for [M + H] 585.2032, found 585.2016.

### Biology

#### Cell culture

The human pancreatic carcinoma MIA PaCa-2 was purchased from China Centre for Type Culture Collection, AsPC-1 and BxPC-3 cell Lines were purchased from Cell Centre of the Institute of Basic Medical Sciences, Chinese Academy of Medical Sciences and Peking Union Medical College. PANC-1 was kindly provided by Nankai Hospital (Tianjin, China). These cancer cell lines were cultured according to the manufacturer’s instruction.

#### Proliferation assays

Cells were planted in 96-well plates at a density of 3000–4000 cells per well and incubated at 37 °C in a humidified CO_2_ incubator for 24 h. Then 3-fold diluted concentrations of tested compound were added respectively which was dissolved by DMSO in the ratio of 1:1000. After indicated incubation period, MTT was added to each well and incubated for 4 h at 37 °C. Then, the supernatant was discarded gently, and the precipitate was dissolved in 100 µL of DMSO in each well. The absorbance (A) at 570 nm was detected with a microplate reader (Multiskan MK3; Thermo Fisher Scientific, MA, USA). Vehicle-treated cells were served as the control for the calculation of relative cell viability (%) of treated group at single concentration. IC_50_ value was calculated directly from the inhibition curve of serial dilution of test compound and was expressed as mean ± SD using the SPSS statistical software (SPSS17.0 Inc., Chicago, IL, USA).

#### Kinase assays

CDK kinase activity (IC_50_ values and inhibitory rate at single concentration) was measured using the kinase profiling services (Shanghai ChemPartner Co., Ltd). Compounds were sent to ChemPartner as a solution in DMSO at concentration of 10 mM.

#### Immunoblots

Lysates were prepared from cells treated for 24 h. Harvested cells were lysed in 20 mmol/L Tris pH 7.7, 100 mmol/L NaCl, 20 mmol/L β-glycerophosphate, 5 mmol/L MgCl2, 1 mmol/L NaF, 0.1% Triton X-100, 5% glycerol plus freshly added 1 mmol/L DTT, 0.1 µmol/L okadaic acid, 1 mmol/L sodium vanadate, and cOmplete Mini Protease Inhibitor Tablets (Roche, #11836170001). For SDS-PAGE, equal amounts of protein/lane were loaded. After gel transfer onto PDV membrane, immunoblots were performed using the following antibodies: CDK9 (Cell Signalling Technology #C12FT; 1:1,000), RNA polymerase II (Abcam #ab5131; 1:1000), MCL-1 (Abcam #Y37; 1:10omogeniospho-Rb (Cell Signalling Technology #d2b12; 1:1,000), c-Myc (Abcam #YB69; 1:1000). Secondary antibodies were from Cell Signalling Technology (#7076 or #7074). ECL detection was performed.

#### Cell cycle and apoptosis analysis

MIA PaCa-2 cells were incubated with test compound at given concentration for indicated time in 6 well plates. The cells were collected and washed with cold PBS for two times. For cell cycle analysis, cells were fixed with 1 ml of 70% ethanol at −20 °C overnight then re-suspended with 400 µL binding buffer at density of 10^6^ cells/mL, with PI staining for 30 min at room temperature. DNA contents were analysed by a BD Accuri C6 flow cytometry (BD Biosciences, Mountain View, CA, USA). For cell apoptosis analysis, after centrifugation and removal of the supernatants, cells were resuspended in 500 µL of 1 ×binding buffer which was then added to 5 µL of annexin V-FITC and incubated at room temperature for 10 min. After adding 10 µL of PI the cells were incubated at room temperature for another 10 min, and their fluorescence intensity evaluated by a BD Accuri C6 (BD Biosciences, Mountain View, CA, USA).

#### Transfection with specific siRNA

Specific siRNAs against CDK9 (Catelog ID: SIGS0004694-1) were purchased from RiboBio (Guangdong, China) together with a scrambled siRNA. For siRNA transfection, cells were transfected with a pooled sample of 3 CDK9-specific siRNA oligos at specific concentrations using riboFECT CP Transfection Kit (Catelog ID: C10511-05, RiboBio, Guangdong, China) according to the manufacturer’s instructions. A scrambled siRNA was applied as a negative control. Approximately 48 h after transfection, the cells were analysed for protein expression by Western blot. The most effective siRNA was used for RNA interference experiment. Statistical analysis was performed using GraphPad Prism (Ver 8.0.2).

#### Molecule docking

The X-ray crystal structure of CDK9 （4BCF) was downloaded from the Protein Data Bank (https://www.rcsb.org). The protein was prepared according to standard operations. The co-crystallized water molecules were deleted, and the “Clean Protein” and “Prepare Protein” tools of Discovery Studio were used to resolve potential problems in protein structures, such as the model missing loop regions, delete alternative conformations, add hydrogen atoms, and generate the protonation state at pH 7.0. The active site was identified according to small molecule ligand and previous reports, and the docking radius of the binding site sphere was set to 9 Å. The compounds were “Full Minimization.” Discovery Studio 4.0 was used to carry out the CDOCKER method to perform docking-based virtual screening.

### Animal studies

#### Compound formulations

Synthetic compound was administered as a suspension or solution in the saline with 5% DMSO and 5% Solutol. GCTB was dissolved in saline. All of the experiments were approved by the Institutional Animal Care and Use Committee of Institute of Medicinal Biotechnology. All experiments were carried out under ethics committee–approved protocols and in compliance with the guidelines for the care of laboratory animals of Institute of Medicinal Biotechnology, CAMS.

#### Oral PK study

SD rats were purchased from Beijing Vital River Laboratory Animal Technology Co., Ltd and randomly divided into three groups (*n* = 3, male). The animals were housed under standard conditions and fastened for 12 h before the treatment. They had free access to water and consumed a standard laboratory diet from 4 h after administration. Blood was taken from the orbital venous plexus at 0, 0.083, 0.25, 0.5, 1, 2, 4, 6, 8, and 24 h after intragastric administration of the test compound (30 mg/kg). The blood samples were collected into heparin anticoagulated EP tubes and centrifuged at 8000 r/min for 5 min at 2−8 °C; the plasma was separated and stored at −70 °C for use. Plasma was centrifuged at 4000 rpm for 10 min at 2−8 °C and stored at −70 °C until analysis. Plasma samples were analysed by LC-MS/MS (AB Sciex API4000 QTRAP) equipped with an ESI ion resource and Analyst 1.5.1 software. The noncompartmental model analysis was used for calculating pharmacokinetic parameters with the WinNolin professional v6.3 program (Pharsight, USA).

#### Mouse xenograft studies

AsPC-1 (5 × 10^7^) cells were injected subcutaneously into 5-week-old female BALB/C nude mice (Beijing Huafukang Bioscience CO. Inc, Beijing, China). After two weeks, mice were omogenizamised and treated with vehicle, gemcitabine (GCTB), or test compound. Blank solvent and test compound was administered by oral gavage (10 ml/kg) with once daily of five days and followed by 2 days off in each week, GCTB was given once a week by 50 mg/kg intraperitoneal injection. Mice were inspected daily for clinical signs, both tumour diameters and body weight were determined 3 times weekly. Tumour volumes were calculated according to the formula “tumor volume (V) = length × width^2^/2”. Median TGI values were calculated as follows: TGI = 100 × [1-(V_treated group at day20_-V_treated group at day1_)/(V_vehicle group at day20_-V_vehicle group at day1_)]. Data were plotted as average with SD in the graphical representation of tumour growth. The *p* values obtained from the two-way ANOVA analysis. Meanwhile, main organs of mice were collected, formalin-fixed and paraffin-embedded for pathological analysis by H&E dyeing. For drug concentration determination, blood and tumour tissues were collected at indicated time after single administration. Standard curve of test compound was established in the acquisition mode of SIM using a SHIMADZU LC-MS instrument. Blood kept in anticoagulant tube coated with heparin sodium was centrifuged to provide the supernatant, which was followed by added with 3-fold acetonitrile to precipitate plasma protein. After centrifugation at 10 × 10^3^ for 5 min, the supernatant was injected directedly into LC-MS instrument for quantification of drug concentration. As for tumour tissues, omogenizationation (Precellys Evolution) was done after weighed accurately and added with 3-fold saline. After centrifugation under same condition as plasma samples, supernatant was separated and injected into LC-MS for quantification.
