# Correction

**DOI:** 10.1080/14756366.2023.2177411

**Published:** 2023-02-08

**Article title:** Design, synthesis and biological evaluation of 2-((4-sulfamoylphenyl)amino)-pyrrolo[2,3-d]pyrimidine derivatives as CDK inhibitors 

**Authors:** Bo Yang, Yanni Quan, Wu-li Zhao, Yingjie Ji, Xiaotang Yang, Jianrui Li, Yi Li, Xiujun Liu, Ying Wang, Yanping Li 

**Journal:** *Journal of Enzyme Inhibition and Medicinal Chemistry*

**Bibliometrics:** Volume 38, Number 1 

**DOI:** https://doi.org/10.1080/14756366.2023.2169282

The authors would like to point out that the wrong version of Table 1 and Figure 3 has appeared in the published article. Right chemical structure of R_2_ group for compound **2g** in Table 1 has been replaced. Also, the missing Figure 3(C) is added. The corrected Table and Figure are presented in the below corrigendum, and does not alter the conclusion drawn from this work. The authors sincerely apologize for these errors during the proof confirmation.

**Figure 3.**
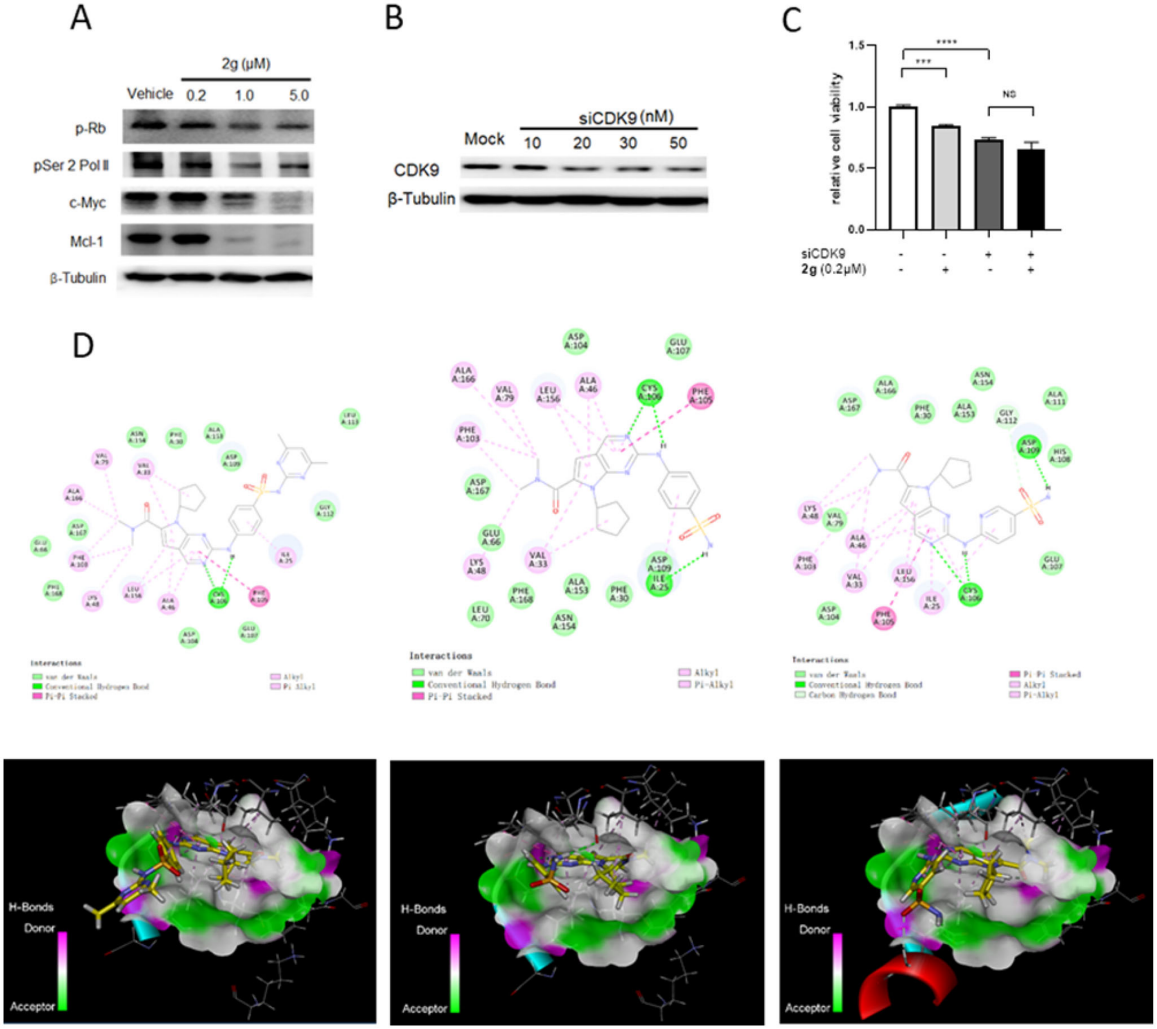
The anti-proliferative activity of new synthetic compounds is mainly mediated by CDK9. (A) **2g** inhibited the phosphorylation of Rb and RNA polymerase II, induced apoptosis through down-regulation of Mcl-1 and c-Myc protein. MIA PaCa-2 cells were incubated with or without **2g** at indicated concentration for 6 h. (B) CDK9 was dose-dependently knockdown by its specific siRNA after 48 h treatment in MIA PaCa-2 cells. (C) Downregulation of CDK9 using siCDK9 (50 nM) decreased the sensitivity of MIA PaCa-2 cell to compound **2g** (0.2 µM). The cells were incubated with **2g** for 72 h. NS no significance; ***p < 0.001; ****p < 0.0001. Whole-cell lysates were subjected to immunoblotting. A representative protein band of three independent experiments is shown. (D) Representative illustration of the binding of synthetic compound to CDK9/cyclin T. Small molecules **1** (left column), **2g** (middle column), and **2i** (right column) were shown in stick representation with carbons colored yellow.

**Table 1.** Anti-proliferative activity of 2-((4-sulfamoylphenyl)amino)-pyrrolo[2,3-d]pyrimidine derivatives in pancreatic cancer MIA PaCa-2 cell culture.

	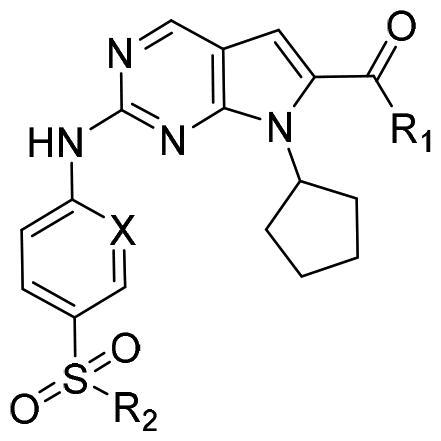			IC_50_ (µM) in MIA PaCa-2^a^	CDK9 inhibitory rate (%) @ 50nM^b^
Cpd.	R_1_	R_2_	X		
**1**	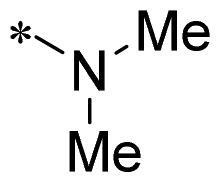	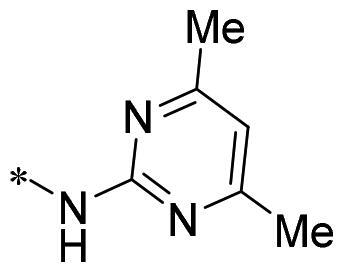	CH	4.98 ± 2.68	98.6
**2a**	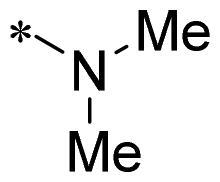	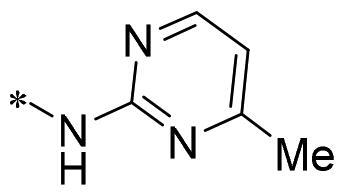	CH	13.00 ± 0.37	100.3
**2b**	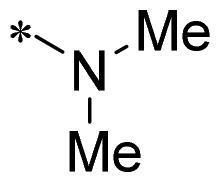	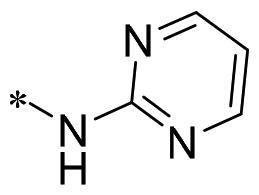	CH	14.35 ± 0.21	98.7
**2c**	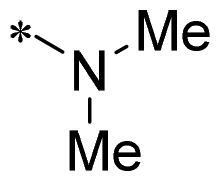	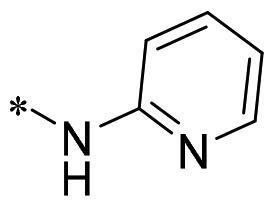	CH	18.80 ± 5.54	99.1
**2d**	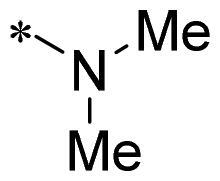	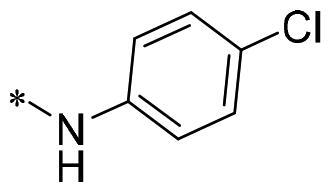	CH	6.23 ± 1.25	91.8
**2e**	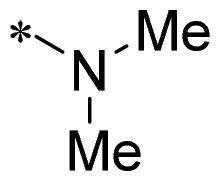	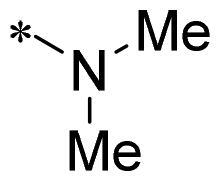	CH	10.50 ± 0.74	89.3
**2f**	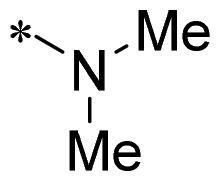	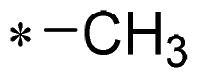	CH	18.90 ± 2.121	96.5
**2g**	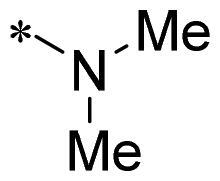	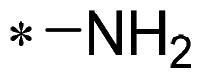	CH	0.52 ± 0.67	97.8
**2h** ^c^	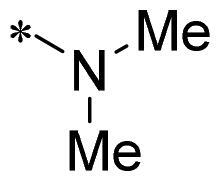	-SO_2_-R_2_ not exist	CH	45.05 ± 16.64	93.2
**2i**	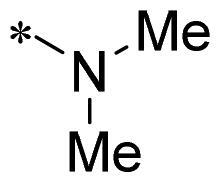	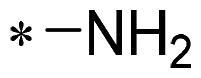	N	>100	27.4
**2j**	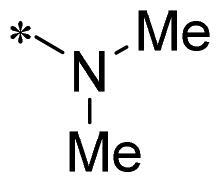	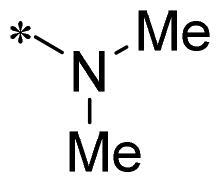	N	69.13 ± 15.51	25.8
**5b**	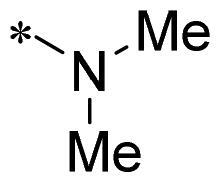	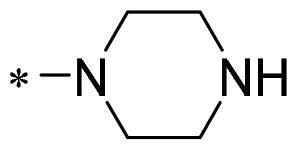	CH	0.94 ± 0.25	98.2
**5c**	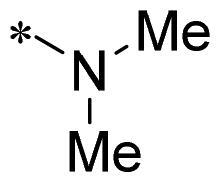	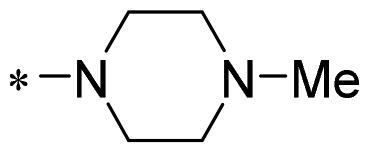	CH	7.80 ± 1.04	99.1
**5d**	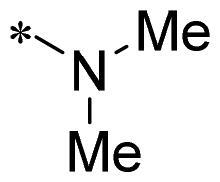	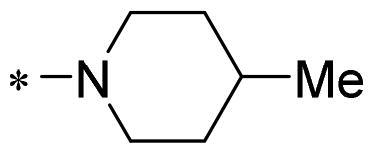	CH	2.85 ± 0.52	80.6
**5e**	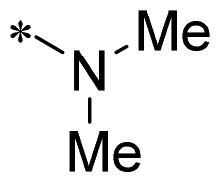	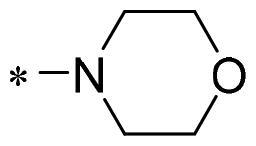	CH	4.35 ± 1.61	81.8
**10a**	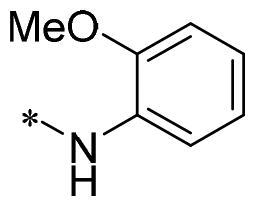	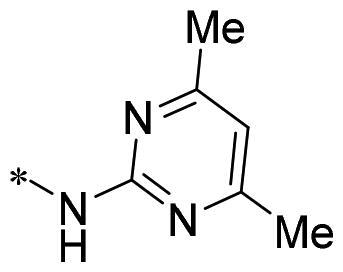	CH	40.76 ± 3.26	−3.1
**10b**	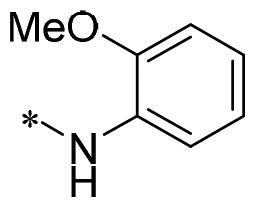	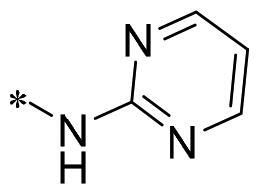	CH	80.76 ± 15.99	54.4
Ribociclib				16.53 ± 7.00	16.9
GCTB				1.04 ± 0.27	

^a^Cell viability was determined after 72 h drug exposure to cells. Compounds **2b**, **2c**, and **5c** precipitated in cell culture medium during incubation. ^b^Test compounds were screened against on CDK9/cyclinT1 by Lance Ultra Assay with ATP concentration at Km. ^c^compound **2h** was previously reported^23^.

